# m^6^A and YTHDF proteins contribute to the localization of select neuronal mRNAs

**DOI:** 10.1093/nar/gkac251

**Published:** 2022-04-19

**Authors:** Mathieu N Flamand, Kate D Meyer

**Affiliations:** Department of Biochemistry, Duke University School of Medicine, Durham, NC 27710, USA; Department of Biochemistry, Duke University School of Medicine, Durham, NC 27710, USA; Department of Neurobiology, Duke University School of Medicine, Durham, NC 27710, USA

## Abstract

The transport of mRNAs to distal subcellular compartments is an important component of spatial gene expression control in neurons. However, the mechanisms that control mRNA localization in neurons are not completely understood. Here, we identify the abundant base modification, m^6^A, as a novel regulator of this process. Transcriptome-wide analysis following genetic loss of m^6^A reveals hundreds of transcripts that exhibit altered subcellular localization in hippocampal neurons. Additionally, using a reporter system, we show that mutation of specific m^6^A sites in select neuronal transcripts diminishes their localization to neurites. Single molecule fluorescent *in situ* hybridization experiments further confirm our findings and identify the m^6^A reader proteins YTHDF2 and YTHDF3 as mediators of this effect. Our findings reveal a novel function for m^6^A in controlling mRNA localization in neurons and enable a better understanding of the mechanisms through which m^6^A influences gene expression in the brain.

## INTRODUCTION

Subcellular RNA localization is an important mechanism of spatial gene expression control in cells. This is especially true in neurons, in which thousands of mRNAs can be localized to distal compartments such as axons and dendrites, where they can in turn undergo local translation to produce proteins critical for axon outgrowth and synaptic plasticity, respectively (1–3). The subcellular trafficking of neuronal transcripts to these distal locations plays important roles in neurodevelopment and synaptic remodeling. However, the mechanisms that govern which mRNAs are destined for subcellular transport are poorly understood. Several RNA binding proteins (RBPs) have been shown to facilitate mRNA transport through their recognition of specific *cis*-acting sequence and structural elements, usually those in 3′UTRs (3–5). However, there is no universal localization element shared by all distally-localized transcripts, and there are likely to be other mechanisms beyond sequence and structure that confer localization information.

In addition to RNA sequence motifs and structural elements, RNA base modifications provide a mechanism for controlling RNA:protein interactions in cells. *N*^6^-methyladenosine (m^6^A) is the most abundant internal mRNA modification, and its levels are particularly high within the brain (6,7). Furthermore, recent studies have revealed important roles for m^6^A in neurodevelopment and learning and memory (8–11), two processes which require precise spatial control of RNA expression (3). m^6^A controls nearly every stage of the mRNA life cycle, and these various functional roles of m^6^A are carried out by m^6^A-dependent regulation of RNA:protein interactions (12,13). In particular, direct recognition of m^6^A residues by YTH domain-containing proteins is a major mechanism through which m^6^A regulates mRNA fate. Several studies have linked YTHDF proteins to mRNA degradation (14–16), but they have also been shown to influence the subcellular localization of methylated mRNAs (17–21). Additionally, m^6^A is enriched in a subset of neuronal mRNAs at the synapse (22). Thus, it is possible that m^6^A serves as a chemical tag for promoting the transport of specific mRNAs to distal neuronal compartments (23). However, this possibility has not been directly explored.

Here, we show that methylated adenosine residues within the 3′UTR promote the localization of a subset of neuronal mRNAs to dendrites and axons (collectively referred to as neurites). Our profiling of the methylomes of neuronal subcellular compartments reveals thousands of m^6^A sites that are enriched in neurites compared to cell bodies. Depletion of the m^6^A methyltransferase, *Mettl3*, alters the neurite localization of hundreds of transcripts, suggesting that m^6^A-mediated RNA localization is likely to be a widely used mechanism within neurons. We further examine two transcripts important for synaptic strength and plasticity, *Camk2a* and *Map2*, and find that m^6^A residues within their 3′UTRs are required for their localization to neurites. In addition, we find that m^6^A-dependent localization of these mRNAs is impaired following YTHDF2 or YTHDF3 reader protein depletion. Collectively, our studies suggest that m^6^A contributes to the localization of a subset of mRNAs within neurons and demonstrate that the *Camk2a* and *Map2* transcripts exhibit m^6^A- and YTHDF protein-dependent neurite enrichment.

## MATERIALS AND METHODS

### Biological resources

#### Cell lines

HEK293T cells were obtained from ATCC and maintained at 37°C and 5% CO_2_, using DMEM (Corning) supplemented with 10% fetal bovine serum (VWR) and Pen/Strep (Gibco).

#### Constructs

3′UTRs for reporter genes, as well as 5xBoxB and 6xMS2 3′UTRs, were cloned downstream of Dendra2 in the pFUGW lentiviral backbone plasmid containing Dendra2 by Gibson assembly. For the 3′UTR reporter assays, we identified m^6^A sites within the 3′UTR by examining antibody-based m^6^A mapping data from neuron and brain samples (6,22,24,25), either by selecting single nucleotide mapped sites or by finding consensus DRACH sequences found within MeRIP-seq peaks. Mutations of m^6^A sites were introduced by either PCR mutagenesis or cloning of gene fragments (IDT) and assembled using Gibson assembly. Cloning of shRNAs was done by ligating annealed oligonucleotides (IDT) into a modified pLKO.1-TRC lentiviral backbone where the puromycin was swapped for a CFP using Gibson assembly. Coding sequences from YTHDF proteins were amplified from a mouse hippocampal cDNA library and cloned into the pFUGW backbone followed by the XTEN linker sequence (26) and λN-HA. A complete list of the oligonucleotides used for cloning can be found in Supplementary Table S7. psPAX2 and pMD2.g were a gift from Didier Trono (Addgene plasmid # 12260 and # 12259). pLKO.1-TRC cloning vector was a gift from David Root (Addgene plasmid # 10878) (27). pET42a-lambdaN+-L+-GSH was a gift from Pascale Legault (Addgene plasmid # 98894) (28). pUbC-nls-ha-stdMCP-stdGFP was a gift from Robert Singer (Addgene plasmid # 98916) (29). pAAV-hSyn-Cre-P2A-dTomato was a gift from Rylan Larsen (Addgene plasmid # 107738). pUCmini-iCAP-PHP.eB was a gift from Viviana Gradinaru (Addgene plasmid # 103005) (30). pAdDeltaF6 was a gift from James M. Wilson (Addgene plasmid # 112867).

#### Virus production

Lentiviral vectors were co-transfected with psPAX2 and pMD2.g plasmids in HEK293T cells (ATCC) using PEI MAX (Polysciences) (31). Viral particles were concentrated by ultracentrifugation on a 20% sucrose cushion and titer was determined in HEK293T cells. AAVs were generated in HEK293T cells as previously described (30) using the PHP.eB capsid and pAdDeltaF6 helper plasmid using PEI MAX. Viral particles were quantified by qPCR using primers in the hSyn promoter and the transfer plasmid as a standard.

#### Primary cell cultures

All experiments were approved by the Duke University Institutional Animal Care and Use Committee. Hippocampal tissue was isolated from single litters of WT or *Mettl3*^flox/flox^ newborn pups (P0.5). Tissue of all animals was pooled and therefore represents a mixture of both sexes. Hippocampi were digested with 0.125% Trypsin (Thermo) in dissection buffer (1× HBSS, 10 mM HEPES, 10 mM MgCl_2_, 10 μM kynurenic acid and 33 mM glucose) for 12 min at 37°C, followed by two washes with dissection buffer containing 1% defined trypsin inhibitor. Tissue was triturated in growth media (Neurobasal-A (Gibco), 1× B27 (Gibco), Pen/strep (Gibco), 0.6 mM Glutatamax (Gibco)) and plated onto coated growth surfaces. Growth medium was changed 1 h after cell plating and Ara-C (Millipore Sigma) was added to 1 μM at DIV3 to prevent growth of glial cells. One-third of the media was subsequently changed twice weekly. For low-density cultures, media was supplemented with 20% of astrocyte-conditioned media. All neurons were maintained at 37°C in 5% CO_2_.

#### Dissociated neuron cultures

For harvesting of RNA and proteins, cells were plated at high density (2.5 × 10^5^ cells per well) on poly-d-lysine (PDL) (Gibco) coated 12-well plates. For imaging experiments, cells were plated at low density (4 × 10^4^ cells per well) on 18 mm #1 round cover glass (EMS) coated with PDL and laminin (Sigma). Growth medium was changed 1 h after cell plating and Ara-C (Millipore Sigma) was added to 1 mM at DIV3 to prevent growth of glial cells. One-third of the media was subsequently changed twice weekly. For low-density cultures, media was supplemented with 20% of astrocyte-conditioned media. To suppress basal synaptic activity, 1 μM TTX and 50 μM D-AP5 (Tocris) was added 16 h prior to cell harvesting. To depolarize neurons, 450 μl of membrane depolarization solution (170 mM KCl, 10 mM HEPES pH 7.4, 1 mM MgCl_2_, 2 mM CaCl_2_) was added for each 1 ml of growth media. For imaging experiments, hippocampal neurons were transduced at a multiplicity of infection (MOI) of 1 at DIV2. For tethering assays, cells were transduced with Dendra2-expressing virus at DIV2 and YTHDF-λN-expressing viruses at DIV4, at a MOI of 1 for each virus. For shRNA-expressing lentivirus, cells were transduced at a MOI of 3 at DIV7. All neurons were maintained at 37°C in 5% CO_2_.

#### RNA isolation from soma and neurites

1–1.5 × 10^6^ dissociated neurons were plated in PET cell culture inserts with 3 μm pores (Millipore) and maintained as high density cultures. Each experiment was performed in duplicate using neurons isolated from distinct litters. For treatment with KCl, neurons isolated from the same litter were used for control and KCl treatment. Wild-type or *Mettl3^flox/flox^* neurons were transduced with 50 000 viral AAV particles (AAV-hSyn-Cre-P2A-dTomato) per cell at DIV7. Neurons were treated with TTX and D-AP5 for 16 h at DIV14. The next day, neurons were either left untreated or were treated with KCl for 2 h at DIV15. Media was removed from both sides of the membrane, and the membrane was rinsed with ice-cold PBS. Neurites were scraped from the bottom of the membrane using a cell scraper and the insert was transferred to a six-well plate containing 1ml PBS, releasing the neurites into the PBS. The soma fraction, which can also contain neurites that fail to extend through the membrane pores, was released from the top of the membrane using a cell scraper and transferred to a 1.5 ml tube. Both fractions were centrifuged at 5000g for 3 min and resuspended in 350 μl of RLT + buffer (Qiagen). Paired samples were isolated without pooling. RNA was purified using QIAGEN RNeasy Plus Mini kit, yielding 50–80 ng and 500–1000 ng from neurite and soma fractions, respectively. RNA quantity and quality were measured using the Qubit RNA high sensitivity assay and the RNA 6000 Pico assay on the Bioanalyzer 2100. Samples with a RIN ≥7 were used for library preparation. Acetone-precipitated proteins from the flowthrough of RNA columns were resuspended in NuPAGE loading buffer for western blot analysis.

### Reagents

#### Western blotting

Protein samples were loaded in a 1 mm thick 4–12% NuPAGE gel (Thermo) and separated at 175V for 60 min. Transfers were carried out at 25 V for 30 min to a nitrocellulose membrane in 2x NuPAGE transfer buffer (50 mM Bicine, 50 mM Bis–Tris, 2 mM EDTA, 10% methanol) using semi-dry transfer. Blocking was carried out for 60 min in 5% nonfat dry milk in 0.1% TBST and antibodies were incubated overnight in 5% BSA (VWR) in 0.1% TBST at 4°C. Secondary antibodies were incubated on membranes in blocking buffer for 1 h at room temperature. ECL reagent (Amersham ECL Prime) was mixed 1:1 and added to the membranes, which were imaged using the Bio-Rad Chemidoc imaging system.

#### Antibodies

The following antibodies and concentrations were used: rabbit anti-METTL3 (Abcam; ab195352; WB-1:2,000, IF-1:300), rabbit anti-Histone H3 (Cell Signaling Technology (CST); 9715;WB-1:2000), rabbit anti-Synapsin I (Millipore Sigma; AB1543; WB-1:1000), rabbit anti-Synaptophysin (Proteintech; 17785-1-AP; WB-1:10 000), rabbit anti-c-Fos (CST; 9F6; WB-1:1000), rabbit anti-GAPDH (Proteintech; 10494-1-AP; WB-1:5,000), mouse anti-β-actin (Genscript; A00702; 1:5000), rabbit anti-YTHDF1 (Abcam; ab252346;WB-1:2000; IF-1,250; IP-2 μg), rabbit anti-YTHDF2 (Abcam; ab246514;WB-1:2000; IF-1250; IP-2 μg), rabbit anti-YTHDF3 (Abcam; ab220161;WB-1:2000; IF-1,250; IP-2 μg), rabbit anti-HA (CST; C29F4; 1:2000; IF-1:500), mouse anti-Phospho-CREB (Ser133) (CST; 87G3; IF-1:800), mouse anti-c-FOS (EnCor; MCA-2H2; IF-500), chicken anti-MAP2 (EnCor Biotech; CPCA-MAP2; IF-1:10 000), rabbit anti-*N*^6^-methyladenosine (abcam, ab151230, dot blot-1:1000), mouse anti-FLAG M2 (Sigma, F1804, WB-1:2000), horseradish peroxidase (HRP)-conjugated goat anti-rabbit (Abcam; ab6721; 1:10 000), HRP-conjugated goat anti-mouse (Invitrogen; 62–6520; 1:10 000), AlexaFluor647-conjugated goat anti-Chicken (Invitrogen; A32933; 1:2000), AlexaFluor647-conjugated goat anti-mouse (Invitrogen; A11001; 1:2000), AlexaFluor546-conjugated goat anti-rabbit (Invitrogen; A-11036; 1:2000), AlexaFluor647-conjugated goat anti-rabbit (Invitrogen; A-21245; 1:2000).

#### smFISH and immunofluorescence

Custom Stellaris^®^ FISH Probes recognizing *Dendra2*, *Map2* and *Camk2a* and labeled with Quasar570, were purchased from Biosearch Technologies. Probe set sequences for *Camk2a* and Dendra2 have been previously described (32,33). For *Actb* and *Shank1*, smiFISH probes were designed with Oligostan (34) and annealed to a dual Quasar570-labeled oligonucleotide. Probe sequences can be found in Supplementary Table S7. smFISH experiments were performed as described (32,34) with some modifications. For reporter assays, DIV7 neurons were used. For endogenous RNA smFISH, DIV14 neurons were used to allow sufficient time for candidate gene expression to begin. Neurons were fixed for 10 min at room temperature with 4% formaldehyde, 4% sucrose in 1× PBS for 10 min, followed by washes (3×) with 1× PBS. Cells were permeabilized in ice-cold 70% ethanol for at least 1 h at 4°C or for with. Cells were re-hydrated in FISH wash buffer (2× SSC, 10% formamide (sigma)) for 10 min. Cells were then incubated with 50 nM Quasar570 labeled mix probe set in hybridization buffer (2× SSC, 10% dextran sulfate, 1 mg/ml *Escherichia coli* tRNA, 20 mg/ml BSA, 2 mM Vanadyl Ribonucleoside complex, 10% formamide) or Stellaris RNA FISH Hybridization Buffer (Biosearch Technologies Cat# SMF-HB1-10) for 16 h at 37°C in a humidified chamber. After two washes for 30 min at 37°C in 2× FISH wash buffer (25 ng/μl DAPI (Millipore Sigma 10236276001) included in second wash), coverslips were mounted using Vectashield Vibrance antifade mounting media (Vector Laboratories, H-1700).

For smFISH/IF, fixed cells were permeabilized with 0.1% triton in 1× PBS for 15 min, followed by 5 min washes (3×) with 1× PBS. Cells were blocked for 30 min with 0.5% UltraPure BSA (Invitrogen AM2616) in 1x PBS before adding primary antibody for 1h at room temperature. Coverslips were washed for 5 min with 1× PBS (3×) and incubated for 1 h with secondary antibody (anti-rabbit Alexa647) for 1 h at room temperature. Following 5 min washes in 1× PBS (3×), cells were fixed in 4% paraformaldehyde, 4% sucrose in 1× PBS for an additional 10 min, followed by additional washes in 1× PBS (3×). Cells were then used for FISH as above.

#### Image acquisition

All images were captured on an inverted wide-field Leica DMi8 microscope equipped with a 63×/1.4 HC PL APO objective, Leica DFC9000 4.2 MP monochrome sCMOS camera, Lumencor SOLA SM light engine, and the following filter cubes: DAPI (EX350/50; DC400; EM460/50), GFP (EX470/40; DC495; EM525/50), RHOD (EX546/10; DC560; EM585/40), and Y5 (EX620/60; DC660; EM700/75). For each cell, a 3D image stack starting below and ending above detectable signal was captured with a z-step size of 210 nm at 12-bit image depth. The resulting image voxel sizes were (*x* × *y* × *z*) 0.105 nm × 0.105 nm × 0.210 nm. For experiments using Dendra2 reporters, Quasar570 signal was obtained first, prior to acquisition of Dendra2 and DAPI signal to avoid possible photoconversion of the Dendra2 protein.

#### RNA-seq, DART-seq and RIP-seq library construction and sequencing

RNA-seq libraries were prepared from 50 ng of total RNA using the NEBNext Single Cell/Low Input RNA library Prep Kit for Illumina and sequenced on an Illumina NovaSeq6000 S-prime flow cell with paired-end 50-bp reads, yielding 40–90 million clusters per library. For DART-seq, 50 ng of RNA was used for *in vitro* deamination as previously described (35) with some modifications. RNA was incubated with 250 ng of recombinant APOBEC1-YTH-HA protein for 4 h at 37 °C in 1x deaminase buffer (10 mM Tris–HCl, pH7.5, 50 mM KCl, 0.1 μM ZnCl_2_). RNA was purified with QIAGEN RNeasy MinElute Cleanup kit and used as input for RNA-seq libraries as above. RIP-seq libraries were prepared from 9 ng of RNA using the SMARTer Stranded Total RNA-Seq Kit v3 – Pico Input Mammalian (Takara) for three biological replicates. For each replicate, the lysates of three wells of neurons grown in a six-well plate were pooled and then equally split for YTHDF1, YTHDF2 and YTHDF3 RIPs. This enabled 3 YTHDF RIPs (one each for YTHDF1,2, and 3) from a single shared input sample. Libraries were sequenced on a single lane of a NovaSeq6000 S-prime with paired-end 50-bp reads, yielding 18–54 million clusters per library.

#### RNA-seq analysis

Sequencing reads were trimmed with Flexbar (v3.5.0) (36) and aligned to the mm10 genome using STAR (v2.7.7) (37) in paired-end mode. Mapped fragments were counted using featureCounts (Subread version 2.0.1) (38) and imported in R for differential gene expression (DGE) analysis using DESeq2 (39). Mitochondrial-encoded RNAs were excluded from analyses as they can be locally synthesized. Genes with a baseMean of less than 10 were excluded from both analyses. For identification of expressed genes in neurites, we considered all RNAs with >5 FPKM. For the DGE analysis of neurite enrichment in WT neurons and changes in expression in *Mettl3* KO cells, we used a threshold of ±1.5-fold change and FDR adjusted *P*-value ≤0.05 for identification of significant changes. For DGE analysis of changes in gene expression following KCl-treatment, we considered reads in the soma fraction only and used a threshold of ±2-fold change and FDR adjusted *P*-value ≤0.05. For differential neurite enrichment analysis, we adapted the ribosome profiling program Xtail (40). Fragment counts from neurite samples were paired with soma sample counts from the same cell culture insert and considered as analogous to ribosome protected fragments (RPFs) and RNA input, respectively, followed by statistical negative binomial modelling. Statistical significance and change in localization (ΔFC_N/S_) were calculated following two pipelines on the normalized counts by (i) assessing the log_2_ fold change in RNA abundance in each compartment across conditions and (ii) comparing the difference in log_2_ ratios of RNAs across compartments. Finally, for each RNA, the pipeline with the highest *P*-value (least significant) was selected to establish the final *P*-value and ΔFC_N/S_. Gene Ontology enrichment analysis was done using the cluster profiler R package (41). Discovery of enriched motifs was performed using STREME (v5.3.3) (42) in RNA mode, comparing neurite-enriched mRNAs to all non neurite-enriched mRNAs for a relative enrichment analysis. For each gene, the sequence of the mRNA isoform with the longest annotated feature probed was selected and retrieved. As a control, sequences of non neurite-enriched mRNAs were similarly obtained. For the 3′UTR comparison, sequences of less than 180 nt were removed from the control set to normalize the average lengths of each sequence set.

#### DART-seq analysis

Sequencing reads were aligned to the mm10 genome using STAR (v2.7.7), followed by duplicate removal and read sorting. To identify high-confidence C-to-U editing sites, we developed Bullseye, a set of custom Perl scripts adapted from HyperTRIBE (43,44). In brief, bam files were parsed with Samtools (v1.11) (45) to build a matrix of coverage at each genomic position. The C2U editing rate in annotated exons and extended 3′UTRs (5 kb) was then compared for each sample against all *Mettl3* KO libraries (eight samples). Sites were called at positions containing a minimum of 50 unique reads, three mutations, 1–95%C2U and at least 1.25-fold increase in %C2U value compared to all individual *Mettl3* KO samples. Only sites found in at least two replicates and residing within a RAC motif were kept. To identify high-confidence sites, the average %C2U value of all biological replicates at called sites was compared in WT and *Mettl3* KO samples, and only sites with at least a 1.5-fold increase in %C2U value relative to *Mettl3* KO samples were selected for downstream analysis. Usage instructions for the Bullseye pipeline is detailed on the project's GitHub repository page: https://github.com/mflamand/Bullseye. To compare %C2U editing at individual sites across conditions and compartments, we fitted the editing frequency in a binomial generalized linear model in R. The significance at each site was then computed using a Wald-test on the fitted data and adjusted for multiple comparisons. The average %C2U at each of these sites was used for visualization in gene tracks. To identify differentially methylated RNAs, we defined a score for each RNA as the cumulative sum of %C2U at all called sites within the RNA. For the analysis of distribution of m^6^A sites across the mRNA landscape, the relative position of sites was calculated using metaPlotR (46), and number of sites or peaks in 5′UTRs, coding sequences, and 3′UTRs were normalized to the median size of each region in their respective enrichment category.

#### RIP-seq analysis

Unique Molecular Identifiers (UMIs) were extracted from read2 using UMI-tools (v1.1.1) (47) with the option: –bc-pattern = NNNNNNN, following by trimming with Cutadapt (v3.4), removing the first six nucleotides of read2 and the adapter sequences. Processed fastq files were aligned to the mm10 genome using STAR (v2.7.7) (37) in paired-end mode. Duplicate reads were removed from the bam files using UMI-tools and unique fragments were counted using featureCounts (Subread version 2.0.1) (38). The count matrix was imported in R for differential gene expression (DGE) analysis using DESeq2 (39) and independent hypothesis weighting (48) for scoring significance of enrichment. Enriched RNAs were defined as those with a fold change in RIP over input of at least 1.25 and an FDR adjusted *P*-value ≤0.05. For pairwise comparisons, we selected differentially enriched RNAs as those displaying at least twice the enrichment (ΔFC ≥ 2) as the compared YTHDF protein.

#### Image analysis

Image stacks of the Quasar570 channel were processed in Leica LasX by 3D blind deconvolution using the AutoQuant algorithm, allowing for resizing to a 16bit-depth and a reduction in background signal. To determine position and to count mRNA molecules in cells, we used FISH-quant (34) software running on MATLAB R2020a (MathWorks). Briefly, to identify outlines of each cell, GFP and DAPI channels were projected in a focus-based approach, and masks were created using CellProfiler (49) (Broad institute, v4.0.7). Masks were then manually curated using Fiji (50) (1.53c) to open ‘holes’ that occur when neurites from the same neuron cross. Outline of the soma was manually defined for each image and was used as nuclei in FISH-quant. The software identified mRNA spots in the 3D stack and non-specific signal was filtered by restricting signal amplitude and width in both *x*–*y* and *x*–*z* axes. Once detection settings were set, detection of mRNAs in all cells was done in an automated manner. Summary data containing the total number of RNAs detected in each cell and in their neurites was exported, and visualization of results was done using R-4.0.3. For representative images presented in figures, the maximal projections of a single image stack for each condition was linearized using Fiji and cropped to display a single neurite. Contrast was adjusted to reduce background signal. The analysis was performed on the uncropped images. All values associated with quantification of smFISH experiments can be found in Supplementary Table S7.

#### RNA immunoprecipitation

For each RIP on endogenous YTHDF proteins, 1.5–2 × 10^6^ hippocampal neurons per well of six-well plates were harvested at DIV14 lysed in RIP buffer (25mM Tris–Cl pH 7.4, 150 mM NaCl, 2.5 mM EDTA, 0.5 mM DTT, 0.5% NP-40, RNase inhibitor, Roche cOmplete mini EDTA-free protease inhibitor) by mixing for 15 min at 4°C. During lysis, 2 μg antibody was added to 20 μl Dynabeads Protein A (Invitrogen) per sample in PBS containing 0.005% Tween and incubated at 21°C for 30 min. Lysate was cleared by centrifugation at 17 000 × g for 10 min and the supernatant was transferred to a new tube and incubated with antibody-coupled beads for 1 h at 4°C. Following immunoprecipitation, beads were washed three times for 5 min with lysis buffer containing 0.1% NP-40. One-fifth of the beads were used for protein elution in 2× NuPAGE buffer with 100 mM DTT, followed by western blot analysis. The rest of the beads were resuspended in 1 ml of Trizol (Invitrogen), and RNA was extracted by adding 200 μl of 1-bromo-3-choloropropane (Millipore Sigma). Precipitated RNA was washed twice with 75% ethanol and resuspended in water. A fraction of the cell lysate was used for RNA input extraction and treated with Turbo DNase (Invitrogen) for 30 min at 37°C. for RT-qPCR analysis, cDNA was produced with Superscript III and Real-Time PCR was performed using iTaq Universal SYBR Green Supermix) in a CFX Connect Real-Time PCR system (Bio-Rad). Enrichment of each RNA was normalized to the input RNA and to the non-methylated RNA *Eif5a*.

For tethering experiments, HEK293T cells were transduced sequentially with lentivirus expressing Dendra2-5xBoxB/6xMS2 and YTHDF-XTEN-λN-HA at a MOI of 3. For each reporter:protein pair, confluent cells were harvested from 2 × 10 cm plates and lysed as above for RIP. Enrichment of each RNA was done by normalization to non-specific binding of Dendra2-reporters in lysate lacking HA tagged proteins.

#### MeRIP-RT-qPCR

For each biological triplicate, 30 μg of total RNA was extracted from HEK293T cells transduced with lentivirus expressing WT or m^6^A-mutant reporters at a MOI of 3 and was fragmented to ∼100 nt fragments using 10x Ambion Fragmentation Reagent for 10 min at 75 °C. Fragments were immunoprecipitated with 5 μg of Rabbit anti-m^6^A antibody (Abcam) precoupled to 50 μl of Protein A/G magnetic beads (Pierce) in 300 μl of immunoprecipitation buffer (50 mM Tris–Cl pH 7.4, 150 mM NaCl, 1% NP-40 substitute, 0.5 mM EDTA) for 1 h at 4°C. Following three washes in IP buffer and in two washes in 0.25× SSPE, RNA was eluted twice with 125uL of elution buffer (50 mM Tris–Cl pH 7.4, 1 mM EDTA, 20 mM DTT, 0.1% SDS) by incubating at 42°C for 5 min. RNA was purified using acidic phenol (Sigma) and chloroform, and ethanol precipitated in the presence of 1 ul of GlycoBlue Coprecipitant (Ambion). RNA was finally resuspended in 10 μl of RNAse free water. cDNA was prepared with Superscript III and random hexamers (Invitrogen) and Real-Time PCR was performed using iTaq Universal SYBR Green Supermix in a CFX Connect Real-Time PCR system (Bio-Rad). To control for immunoprecipitation efficiency, the enrichment of WT and mutant reporters pulled down in each replicate was normalized to the enrichment of an endogenous m^6^A site found in *ACTB*.

#### Quantitative PCR

cDNA was prepared with iScript Reverse Transcription Supermix (Bio-Rad) using 10 ng of total RNA and then diluted 1:5 with nuclease-free water. 2 ul of diluted cDNA was used per 10 μl reaction (in triplicate) with iTaq Universal SYBR Green Supermix (Bio-Rad) in a CFX Connect Real-Time PCR system for 40 cycles, followed by a melt curve analysis. Primers and their respective amplification efficiencies are listed in Supplementary Table S7. Changes in expression were calculated with the ΔΔCT method (51), accounting for primer efficiency.

#### Immunofluorescence

Neurons were fixed for 10 min at room temperature with 4% formaldehyde, 4% sucrose in 1× PBS for 10 min, and permeabilized with 0.1% triton in 1× PBS for 15 min. Cells were blocked for 1 h with 5% BSA (VWR) in 1× PBS before overnight incubation with primary antibody at 4°C. Coverslips were washed with 1× PBS (3×) and incubated for 1 h with secondary antibody at room temperature. Coverslips were washed in 1× PBS for 5 min (3×), including 25 ng/μl DAPI in the second wash. Coverslips were mounted using Vectashield Vibrance antifade mounting media.

#### m^6^A dot blot

To purify poly(A) RNA, total RNA was subjected to two rounds of poly(A) selection using the Dynabeads mRNA Purification Kit (Invitrogen). 50 ng/μl of poly(A) purified RNA was heat denatured in TE buffer for 2 min at 95°C followed by immediate chilling on ice for 3 min. 100 ng of denatured RNA was dotted on a BrigtStar-Plus positively charged nylon membrane (Invitrogen) and immediately crosslinked in a Stratalinker UV 2400 (Stratagene) using the autocrosslink mode. After crosslinking, the membrane was washed for 5 min in 0.1% TBST and stained with methylene blue. Methylene blue staining of the membrane was imaged with the Bio-Rad Chemidoc imaging system. The membrane was then blocked for 1 h in 5% nonfat dry milk in 0.1% TBST and incubated with primary rabbit anti-m^6^A antibody (1:1000, Abcam) in 0.1% TBST for 1 h at 21°C. Following extensive washing with 0.1% TBST, HRP-conjugated goat anti-rabbit IgG was diluted 1:10 000 in 0.1% TBST and added to the membrane for 1 h at 21°C. The membrane was washed again in 0.1% TBST three times for 5 min, developed with ECL reagent (Amersham ECL Prime) and imaged using the Bio-Rad Chemidoc imaging system. Intensities of methylene blue staining and m^6^A signal were quantified with Image Lab software (BioRad, v6.1).

### Statistical analyses

The details about particular statistic parameters and data representation are specified in the figure legends. All statistical analyses were performed using R-4.0.3. Significance of the changes in mRNA localization by smFISH were measured using a two-way Wilcoxon-test. For measurement of endogenous mRNAs, a two-way Wilcoxon-test was used. When multiple comparisons were performed, *P*-values were adjusted using the false discovery rate (FDR) method. A summary of values and statistics of the smFISH quantification data used to generate graphs can be found in Supplementary Table S7. For bar graphs, data are represented as the mean ± S.D. For RIP-RT-qPCR analysis, significance was measured using a Student's *t*-test adjusted for multiple comparisons using the FDR method. Differences in RNA abundance and neurite enrichment for methylated and non-methylated RNAs were measured using a Mann–Whitney *U* test. For the relative enrichment of editing sites across the mRNA landscape, significance was measured with a chi-square goodness of fit test. For all analyses, a value of *P* ≤ 0.05 was assumed to be significant.

## RESULTS

### Identification of the local transcriptome of hippocampal neurons

**Figure 1.**
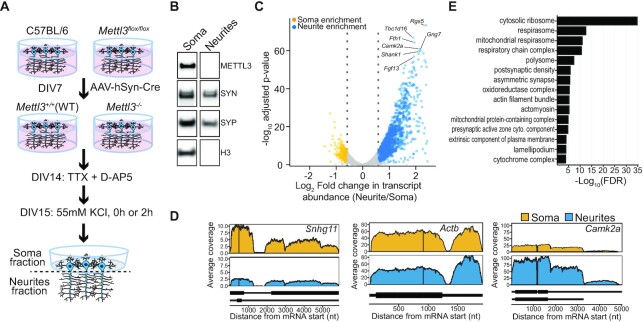
Characterizing the local transcriptome of cultured hippocampal neurons. (**A) E**xperimental design for isolation of soma and neurites. Hippocampal neurons isolated from C57BL/6 (WT) or *Mettl3^flox/flox^* animals are plated on a microporous membrane and transduced with AAV-hSyn-Cre at DIV7. At DIV14, synaptic activity is silenced with TTX and D-AP5. At DIV15, neurons are subjected to KCl-induced depolarization for 0 or 2 h. Soma and neurite fractions are then isolated from the top and bottom of the membrane, respectively. (**B**) Western blot analysis of protein extracts from soma and neurite fractions. Nuclear proteins METTL3 and H3 are secluded from neurites. Pre-synaptic proteins SYN and SYP are found in both soma and neurite samples. (**C**) Volcano plot shows transcripts enriched and depleted in neurites relative to the soma. *Blue*, neurite-enriched genes (FC ≥ 1.5, FDR ≤ 0.05). *Yellow*, neurite-depleted genes (FC ≤ 1.5, FDR ≤ 0.05). The top 7 most significantly enriched genes are highlighted. (**D**) Normalized coverage tracks of representative mRNAs displaying neurite depletion (*Snhg11*), expression in neurites but no enrichment (*Actb*), and neurite enrichment (*Camk2a*). (**E**) False discovery rate for the top 15 overrepresented cellular compartment gene ontology terms for neurite-enriched RNAs. See also Supplementary Figure S1.

To identify RNAs that localize to axons and dendrites, we cultured mouse hippocampal neurons on a microporous membrane, which enables physical separation of dendrites and axons from the cell body (soma) (52–54) (Figure 1A). This method results in a clear separation of the two compartments, as shown by the presence of nuclear proteins exclusively in the soma fraction and synaptic proteins in both fractions (Figure 1B). We extracted RNA from the soma and neurites of mature (DIV 15) neurons and used RNA-seq to identify RNAs from each compartment. To identify transcripts enriched in neurites relative to the cell body, we used differential gene expression analysis (39) to establish an enrichment ratio which calculates the fold change in RNA abundance in the neurite and soma fractions (FC_N/S_). We identified 2420 RNAs that were significantly enriched in neurites relative to cell bodies, and 657 RNAs that were enriched in cell bodies relative to neurites (Figure 1C; Supplementary Table S1). RNAs that have previously been shown to localize to dendrites (55), such as *Camk2a and Shank1*, were among the most enriched transcripts in neurites (Figure 1C and D; Supplementary Table S1). In contrast, RNAs that primarily localized to the nucleus, such as the small nucleolar RNA host gene *Snhg11*, were found almost exclusively in the soma fraction (Figure 1D; Supplementary Table S1). In addition, we observed a high degree of overlap between transcripts detected in hippocampal neurite samples and those found in the neurites of Ascl1-induced neurons (54), cortical neurons (53) and mouse hippocampal neuropil samples (56), indicating the accuracy of our approach (Supplementary Figure S1A).

The majority of transcripts that we identified as enriched in neurites were protein-coding genes (64.1%) (Supplementary Figure S1B). Many of these mRNAs encoded proteins found in the postsynaptic density, as well as proteins involved in mitochondrial and ribosomal functions (Figure 1E, Supplementary Figure S1C). Additionally, neurite-enriched mRNAs had slightly longer 3′UTRs compared to non-enriched mRNAs or soma-enriched mRNAs (Supplementary Figure S1D), which is consistent with previous reports of long 3′UTR isoforms enriched in the hippocampal neuropil (53,57,58).

Motif analysis of neurite-enriched mRNAs revealed the presence of G-rich and G-tetrad ((GGC)_N_) sequences, which are frequently found in G-quadraplex structures (59) (Supplementary Figure S1E). These motifs were not identified when we restricted our analysis to the 5′UTR, coding sequence (CDS), or 3′UTR regions alone, suggesting that these motifs are not enriched within a particular region of neurite-localized transcripts (Supplementary Figure S1E). G-quadraplexes have been linked to translational repression (59–61) and mRNA transport (62,63); however, whether these structures occur more frequently in neurite-localized transcripts and what roles they may play are currently unknown.

In addition to mRNAs, we also detected several noncoding transcripts in hippocampal neurites. This includes long noncoding RNAs (lncRNAs) traditionally thought to localize to the nucleus, such as *Malat1, Neat1* and *Meg3* (64). Although these transcripts were readily detected in neurites, they were present at lower levels than in the soma and therefore were not classified as being neurite-enriched (Supplementary Table S1). A similar distribution of these lncRNAs has been reported in the soma and dendrites of hippocampal neurons (65). Other lncRNAs, such as the cytoplasmic lncRNAs *Bc1* and *Norad*, were also found in neurites, albeit at similar levels as in the soma (Supplementary Table S1). Interestingly, both *Bc1* and *Norad* have been linked to synaptic function and morphology (66–68).

### Membrane depolarization alters neurite enrichment of a subset of RNAs

**Figure 2.**
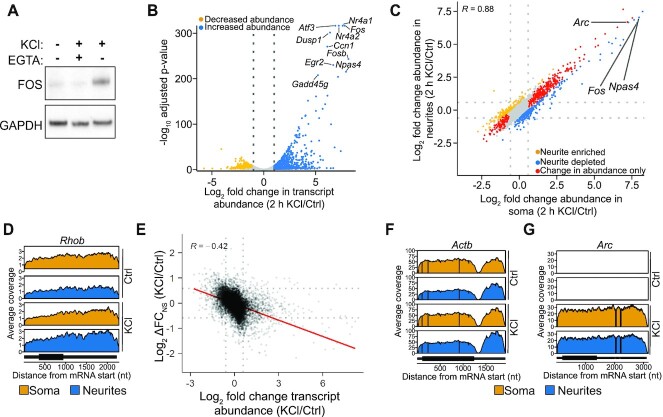
Membrane depolarization alters the localization of a subset of neuronal transcripts. (**A**) KCl treatment induces FOS expression in a calcium-dependent manner. Hippocampal neurons were treated with 55 mM KCl in the presence or absence of 2.5 mM EGTA for 2 h. Western blot shows KCl-dependent c-FOS induction which is abolished with EGTA. (**B**) Volcano plot showing changes in mRNA abundance following KCl treatment. Ctrl: cells treated with D-AP5 and TTX for 16 h. KCl: cells additionally treated with KCl for 2 h. *Blue*, KCl-induced genes (FC ≥ 2, FDR ≤ 0.05). *Yellow*, KCl-depleted genes (FC ≤ −2, FDR ≤ 0.05). Top 10 most induced genes are highlighted. (**C**) Fold change in transcript abundance in the soma and neurites after KCl treatment. *Yellow*, transcripts with increased enrichment in neurites (FC_N/S_ > 1.5, *P*≤ 0.05, *n* = 339). *Blue*, transcripts with decreased enrichment in neurites (FC_N/S_ ≤ −1.5 and *P*≤ 0.05, *n* = 516). *Red*, transcripts with changes in mRNA abundance but no change in enrichment (|FC| ≥ 1.5, *n* = 546). Pearson correlation for fold change in KCl/Ctrl mRNA ratios is indicated. Dotted lines: Log_2_FC thresholds. (**D**) Normalized coverage tracks of *Rhob* which displays increased neurite enrichment following KCl treatment. (**E**) Scatterplot showing a negative correlation between changes in RNA abundance and changes in neurite enrichment following KCl treatment. Pearson correlation coefficient for FC_N/S_ and ΔFC_N/S_ is indicated. Trendline for data is indicated in red. Dotted lines indicate ± Log_2_(1.5). (F, G) Normalized coverage tracks for *Actb* (**F**), and *Arc* (**G**). See also Supplementary Figure S2.

Previous studies have shown that synaptic activity can induce the dendritic localization of select neuronal RNAs (69–75). However, the effects of synaptic activity on RNA localization on a global scale have not been examined. Therefore, we sought to measure changes in RNA enrichment in neurites following neuronal activation. To do this, we subjected TTX- and D-AP5-silenced neurons to 2 h of sustained KCl-induced membrane depolarization (76–78) and isolated RNA from the soma and neurite fractions (Figure 1A). As expected, this treatment led to the calcium-dependent induction of the immediate early gene (IEG) c-*Fos*, as well as phosphorylation of CREB (Ser133), and strong induction of *Fos*, *Npas4*, and *Arc* mRNA levels (Figure 2A, Supplementary Figure S2A and B). We then subjected RNA extracted from the soma and neurites of KCl-treated neurons to RNA-seq and performed differential gene expression analysis. We found 1,112 transcripts that were upregulated following KCl treatment, including several IEGs known to be elevated following synaptic activity (78), such as *Fos*, *Npas4*, *Gadd45g* and *Arc* (Figure 2B; Supplementary Figure S2C and D; Supplementary Table S2).

We next analyzed RNA enrichment in the neurites of KCl-treated neurons. We identified 1569 transcripts that were enriched in neurites (FC_N/S_) following KCl treatment, the majority of which (81.7%) were also enriched in neurites in untreated cells (Supplementary Figure S2E and F; Supplementary Table S2). To identify transcripts whose localization was significantly altered following KCl treatment, we calculated the difference in neurite enrichment of transcripts in stimulated and unstimulated cells (ΔFC_N/S_). To do this, we normalized gene expression in each neurite sample to its paired soma sample to account for the changes in gene expression that accompany KCl treatment, allowing us to identify changes in RNA localization between conditions that are not biased by large changes in gene expression.

Our analysis identified 339 transcripts whose localization to neurites increased after KCl treatment (Figure 2C and D; Supplementary Table S3). We found that ΔFC_N/S_ was negatively correlated with changes in RNA expression in the soma (*R*_Pearson_ = −0.42; Figure 2E), suggesting that strongly induced RNAs are less likely to exhibit activity-dependent increases in neurite localization. Importantly, these observations were not caused by a global change in RNA abundance in each compartment (Supplementary Figure S2G). Surprisingly, we did not find that KCl treatment significantly enhanced the neurite enrichment of *Camk2a*, *Map2*, *Actb* and *Arc*, which have been previously shown to exhibit activity-dependent dendritic localization (69–72,74,75). This could be due to the low magnitude of the reported increase in neurite localization following synaptic activity for some of these transcripts (1.1–1.2*-*fold for *Camk2a* and *Map2* (71,79), which remains below our significance thresholds). Indeed, we observed a modest increase in neurite enrichment for *Actb*, but this also remained below our thresholds for significance (Figure 2F, Supplementary Table S3). *Arc* was also not significantly enriched in neurites following KCl treatment. However, compared to other IEGs such as *Npas4* and *Fos*, which are depleted from neurites of KCl-treated neurons, *Arc* was abundantly expressed in neurites after KCl treatment (Figure 2C and G, Supplementary Figure S2H). This suggests that a portion of newly transcribed *Arc* localizes to neurites, as previously reported (74), but that this is an intrinsic property of the *Arc* transcript, and not due to the increased localization of an existing pool of RNA. Altogether, we found that neurite enrichment of RNAs was strongly correlated between untreated and KCl-treated neurons (*R*_pearson_ = 0.88) (Figure 2C, Supplementary Figure S2I). These results suggest that, although KCl-mediated neuronal activation enhances the localization of select transcripts to neurites, it may not be a major driver of mRNA transport to neurites in cultured hippocampal neurons. Instead, both the mRNA expression level and intrinsic *cis*-acting elements likely play a greater role in neurite localization.

### Loss of *Mettl3* alters neurite enrichment of a subset of mRNAs

**Figure 3.**
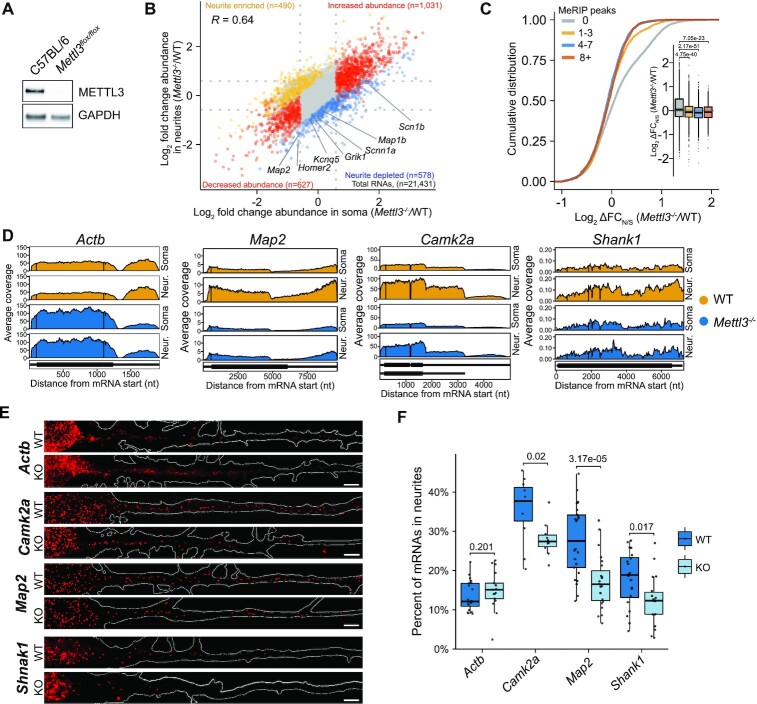
Loss of *Mettl3* alters mRNA localization in neurons. (**A**) Western blot shows METTL3 depletion in cultured hippocampal neurons from *Mettl3*^flox/flox^ mice. WT or *Mettl3^flox/flox^* neurons were treated with AAV-hSyn1-Cre-P2A-dTomato at DIV7 and protein was collected at DIV14. (**B**) Broad changes in neurite enrichment are observed in *Mettl3*-depleted neurons. Fold change in transcript abundance in the soma and neurites of *Mettl3^–/–^* neurons is shown. *Yellow*: transcripts with increased enrichment in neurites (FC_N/S_ ≥ 1.5, *P*-value ≤ 0.05, *n* = 490). *Blue*: transcripts with decreased enrichment in neurites (FC_N/S_ ≤ −1.5 and FDR ≤ 0.05, n = 578). *Red*: transcripts with changes in mRNA abundance but no change in enrichment (FC ≥ 1.5). Pearson correlation for the change in *Mettl3^–/–^* neurons relative to WT neurons is indicated. Dotted lines: Log_2_FC thresholds. A subset of RNAs with known function in neurites and synapses are highlighted. (**C)** The number of MeRIP-seq peaks within a transcript is associated with decreased neurite enrichment in *Mettl3^–/-^*cells. Shown are cumulative distribution plots and boxplots (insets) of fold change in neurite enrichment (ΔFC_N/S_) in *Mettl3^–/–^*/WT for transcripts with 0, 1–3, 4–7 or >8 MeRIP-seq peaks. RNAs with baseMean ≥100 are included; *n* = 8077 (0 peaks); *n* = 3885 (1–3 peaks); *n* = 2043 (4–7 peaks); *n* = 1052 (8 + peaks). *P*-values: result of a two-sided Mann–Whitney *U*-test. (**D)** Normalized coverage tracks of compartment-specific RNA-seq data for *Actb*, *Map2* and *Camk2a* in *Mettl3^–/–^* neurons. (**E**) Representative images showing smFISH signal (red) detecting endogenous *Actb* (control), *Camk2a*, *Map2* and *Shank1* in WT and *Mettl3* KO neurons. Neuron outline is shown in white. Scale bars, 5 μm. (**F**) Boxplots of the quantification of mRNA localization in neurites for endogenous *Actb*, *Camk2a, Map2*, and *Shank1* as measured by smFISH. Individual values for each cell are plotted as gray dots. A two-way Wilcoxon-test was used to test significance. See also Supplementary Figure S3.

Previous studies have identified several sequence elements in mRNA 3′UTRs that confer axonal or dendritic localization in neurons (3,80,81). Although some transcripts harboring these elements were identified in our neurite-enriched transcriptome, these sequence motifs were not a universal feature of all mRNAs localized to neurites (Supplementary Figure S1E). In addition to RNA sequence, methylation of adenosine residues (m^6^A) is a feature of many 3′UTRs which has been shown to influence the subcellular localization of mRNA (17–21). Intriguingly, we identified the m^6^A consensus motif (DRACH; D = A,G,U; R = G,A; H = A, C, U) as a weakly, albeit significantly, enriched sequence within the CDS and 3′UTR of neurite-enriched mRNAs compared to non-enriched mRNAs (Supplementary Figure S1E). We therefore wondered whether m^6^A contributes to mRNA localization to neurites. To investigate this, we cultured hippocampal neurons from *Mettl3*^flox/flox^ mice on microporous membranes as above to separate neurite and soma fractions. Viral delivery of Cre recombinase led to efficient targeting of the *Mettl3* locus and near complete removal (>95%) of METTL3 protein in cultured neurons, as well as reduced m^6^A levels in poly(A) purified RNA (Figures 1A, 3A, Supplementary Figure S3A–C).

At the level of overall gene expression, *Mettl3* knockout (KO) caused the upregulation of 1039 transcripts and downregulation of 736 transcripts (Supplementary Figure S3D, Supplementary Table S4). 37.7% of upregulated and 47.9% of downregulated RNAs overlapped with methylated transcripts identified in the mouse brain by MeRIP-seq (6,22) or m^6^A-CLIP/IP (24). Surprisingly, we found no positive correlation between the increase in mRNA abundance in *Mettl3* KO cells and the number of MeRIP-seq peaks (6), and instead found that mRNAs with the most MeRIP-seq peaks (>8 peaks) showed reduced abundance in *Mettl3* KO neurons (Supplementary Figure S3E).

We next assessed whether *Mettl3* depletion alters RNA enrichment in neurites. We compared FC_N/S_ in wildtype (WT) and *Mettl3* KO neurons and identified 578 transcripts with decreased neurite enrichment in *Mettl3* KO neurons and 490 transcripts with increased neurite enrichment (Figure 3B; Supplementary Table S4). RNAs depleted from neurites after *Mettl3* KO were mostly protein-coding genes (437 RNAs; 75.6%) and preferentially encoded proteins found at synapses and in neuronal projections (Figure 3B, Supplementary Figure S3F and G). In contrast, RNAs with increased neurite enrichment in *Mettl3* KO neurons included a higher proportion of non-coding RNAs and pseudogenes (208 RNAs; 42.4%), and the mRNAs in this group did not encode proteins associated strongly with any particular GO category. In addition, the majority of RNAs (67.7%) exhibiting a *Mettl3*-dependent reduction in neurite enrichment showed evidence for methylation (6,22,24), and RNAs with MeRIP-seq peaks (6) were significantly more likely to have reduced neurite enrichment in *Mettl3* KO neurons compared to non-methylated RNAs (Figure 3C). Together, these data indicate that *Mettl3* depletion reduces the enrichment of a subset of transcripts in hippocampal neurites and suggest that these effects are due at least in part to the loss of m^6^A.

### Loss of *Mettl3* reduces neurite localization of select methylated RNAs

We next sought to use an orthogonal approach to validate the effects of *Mettl3* depletion on RNA localization. To do this, we performed single-molecule FISH (smFISH) on endogenous mRNAs in WT and *Mettl3* KO neurons (Supplementary Figure S3H and I). We selected three mRNAs for validation which exhibited different levels of reduced neurite enrichment after *Mettl3* depletion: *Map2* and*Shank1*, which showed a 50% and 40% reduction in neurite enrichment, respectively, and *Camk2a*, which showed a more modest 20% reduction in neurite enrichment (Figure 3D; Supplementary Table S4). These transcripts also encode proteins important for synaptic function (82–86). As a control, we selected *Actb*, a transcript with no change in neurite enrichment in *Mettl3^–^^/^^–^* neurons (Figure 3D).

We then used smFISH to visualize endogenous *Map2*, *Camk2a*, *Shank1* and *Actb* mRNAs in WT and *Mettl3* KO neurons (Figure 3E). Single mRNA molecules were identified using FISHquant (34), and both the absolute number of mRNAs localized to neurites as well as the percentage of mRNAs localized to neurites (# mRNA molecules in neurites/# total mRNA molecules in the neuron) was determined (Supplementary Figure S3J; see Methods). Consistent with what we observed in neurons grown on microporous membranes, the percentage of *Map2, Shank1* and *Camk2a* transcripts in neurites was reduced in *Mettl3* KO neurons, whereas *Actb* was unchanged (Figure 3E and F). Furthermore, the magnitude of the reduction observed for *Map2, Shank1* and *Camk2a* as assessed by smFISH was similar to what was observed by RNA-seq (40%, 35% and 27% reduction in the percentage of mRNAs in neurites, respectively). Together, these data validate the results of our transcriptome-wide analysis and demonstrate that *Mettl3* contributes to the neurite localization of select mRNAs.

### Mutation of 3′UTR m^6^A sites alters the localization of *Camk2a* and *Map2* mRNAs to neurites

**Figure 4.**
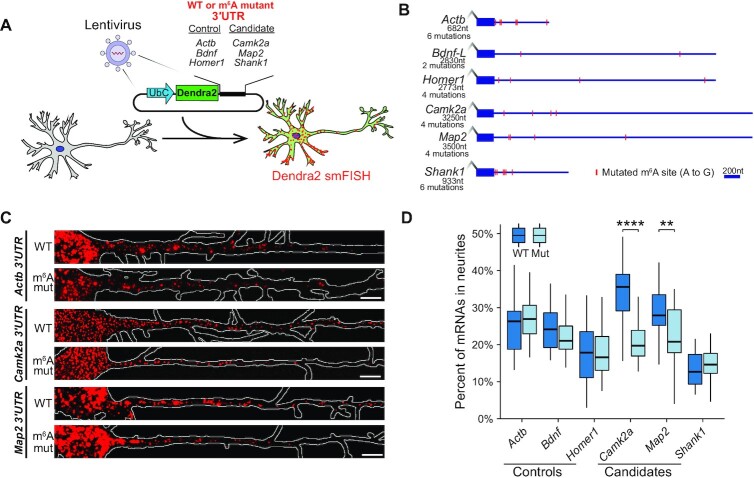
m^6^A-dependent mRNA localization in primary neurons. (**A**) Overview of the mRNA reporter assay. Hippocampal neurons were transduced with lentivirus expressing a reporter mRNA containing the Dendra2 CDS followed by the 3′UTR from a select mRNA. 3′UTRs contained either the wild-type sequence (WT) or A to G mutations at m^6^A sites (m^6^A mutant). Reporter mRNA localization was assessed by smFISH targeting the Dendra2 CDS. (**B**) Shown are the 3′UTRs cloned downstream of Dendra2 in reporter mRNAs and positions of m^6^A sites mutated in each reporter (red). The length of each 3′UTR and the number of mutations is indicated for each reporter. (**C**) Representative images of WT and m^6^A mutant reporter smFISH for *Actb, Camk2a* and *Map2*. Neuron outline is shown in white. Scale bars, 5 μm. (**D**) Boxplots of the quantification of smFISH data showing the percentage of total Dendra2 mRNAs localized to neurites for each reporter. *P*-values calculated by two-sided Wilcoxon-test. *P < 0.05, ****P < 0.0001. See also Supplementary Figure S4.

Our transcriptome-wide analysis indicates that *Mettl3* depletion in neurons alters the localization of hundreds of mRNAs to neurites (Figure 3B). However, to rule out potential indirect effects of *Mettl3* KO and specifically examine the impact of m^6^A, it is necessary to directly disrupt m^6^A sites and assess the effects on mRNA localization. To achieve this, we established an mRNA reporter system which enables us to directly assess the effect of individual m^6^A sites on mRNA localization. In this system, a 3′UTR of interest is placed downstream of the Dendra2 CDS. The reporter is then introduced via lentiviral delivery into cultured mouse hippocampal neurons, and smFISH using Dendra2-targeting probes is performed to enable precise quantification of reporter mRNA localization (Figure 4A). To determine the effect of m^6^A sites within the 3′UTRs of *Camk2a*, *Map2* and *Shank1* on mRNA localization, we generated two versions of the reporter mRNA for each 3′UTR: one in which the wild-type sequence was used (WT) and another in which all m^6^A sites were mutated to G to preclude methylation (m^6^A mutant) (Figure 4B; Supplementary Table S7). MeRIP-RT-qPCR was used to confirm that A-to-G mutation reduced m^6^A levels in regions surrounding m^6^A sites in each reporter (Supplementary Figure S4A and B). As controls, we also generated reporter mRNAs for three transcripts (*Actb*, *Bdnf* and *Homer1*) which contain m^6^A sites, but which did not show *Mettl3*-dependent changes in localization (Figure 3B).

We next expressed each individual reporter mRNA in cultured hippocampal neurons. After 7 days, neurons were fixed and subjected to smFISH targeting the Dendra2 CDS, and both the absolute number and the percentage of mRNAs localized to neurites was determined as above (Figure 4C and D; Supplementary Figure S4C and D). We found that the different WT 3′UTRs conferred variable levels of neurite localization, from as low as 12% (*Shank1*) to as high as 36% (*Camk2a*) (Figure 4D). We then analyzed whether mutation of m^6^A sites altered reporter mRNA localization. For *Camk2a* and *Map2*, we found that 3′UTR m^6^A mutation led to a decrease in reporter localization to neurites (Figure 4C and D). Interestingly, mutation of m^6^A sites within the *Shank1* 3′UTR failed to alter reporter mRNA localization (Figure 4D). It is possible that m^6^A residues within other regions of *Shank1* mediate the altered localization that we observed after *Mettl3* depletion (Fig. 3F). Indeed, *Shank1* contains m^6^A sites within both the 5′UTR and CDS in addition to the 3′UTR (6,7,22,24,25). Additionally, the *Shank1* 3′UTR sequence generally drove the localization of the reporter mRNA to neurites less efficiently than the others we tested, again suggesting that elements outside of the 3′UTR may also contribute to neurite localization (Figure 4D). Importantly, none of the control 3′UTRs we tested showed evidence for m^6^A-dependent neurite localization, which is consistent with our data from *Mettl3* KO neurons. Together, these results indicate that 3′UTR m^6^A sites can directly contribute to the localization of mRNAs to neurites.

### m^6^A is abundant in neurite-enriched mRNAs

To gain further insights into the role of m^6^A in RNA localization, we next sought to measure the methylation levels of transcripts localized to distinct neuronal compartments. This has traditionally been difficult to do, since the requirement of most m^6^A mapping methods for high amounts of input RNA precludes the profiling of low-input samples such as those obtained from neurites. To overcome this issue, we employed DART-seq, an approach recently developed by our laboratory which enables transcriptome-wide m^6^A mapping from ultra-low amounts of input RNA (35). DART-seq utilizes a fusion protein consisting of the m^6^A-binding YTH domain tethered to the cytidine deaminase, APOBEC1, to direct C-to-U (C2U) editing at cytidines that invariably follow m^6^A sites. C2U editing events and C2U editing rates (%C2U) can then be used to identify and quantify m^6^A transcriptome-wide (35).

**Figure 5.**
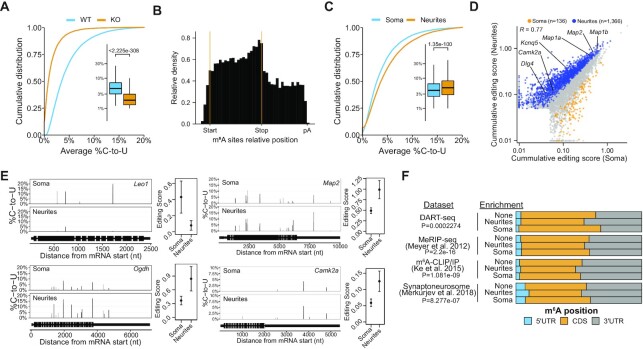
DART-seq identifies condition- and compartment-specific m^6^A sites. (**A)** Cumulative distribution plot and boxplots (insets) of the %C2U values for all DART-seq sites in WT and *Mettl3* KO samples. A two-way paired Wilcoxon test was used to test significance. (**B**) Metagene analysis showing a histogram of the distribution of called sites identified by DART-seq. (**C**) Cumulative distribution plot and boxplots (insets) of %C2U in soma and neurites. A two-way paired Wilcoxon test was used to test significance. (**D)** Scatterplot of the RNA editing score in soma and neurite samples. RNAs with higher editing in neurites are colored in blue, RNAs with higher editing in the soma are colored in yellow. Significance is the result of a Wald-test, FDR < 0.05. *n* = 5643. Pearson correlation of scores is indicated. (**E**) Gene tracks showing examples of RNAs with increased average %C2U editing in the soma (*Leo1*) or neurites (*Ogdh, Map2, Camk2a*). The editing score for each mRNA is plotted on the right of the gene tracks as the mean ± standard error. *N* = 4. (**F**) m^6^A sites are enriched in the 3′UTR of neurite-localized mRNAs. The proportion of m^6^A sites identified by DART-seq, MeRIP-seq(6), m^6^A-CLIP/IP (24) or MeRIP-seq (22) within the 5′UTR, CDS and 3′UTR is shown for mRNAs enriched in the soma or neurites. A chi-squared test was used to compare the expected distribution of sites in each region. Expected number of sites was length-normalized by adjusting to the median length of the 5′UTR, CDS and 3′UTR in each mRNA enrichment category. See also Supplementary Figure S5.

We extracted neurite and soma RNA from hippocampal neurons and subjected each fraction to *in vitro* DART-seq. In parallel, we performed the same experiment using neurite and soma RNA from *Mettl3* KO neurons. We then used Bullseye, a computational pipeline we recently developed for DART-seq analysis (44), to identify C2U editing events that occur in WT RNA samples but are depleted in *Mettl3* KO samples (Figure 5A; Supplementary Figure S5A). We employed additional filters to include only the sites found in multiple biological replicates and within the m^6^A consensus sequence (RAC), which resulted in a list of 18 231 m^6^A sites in 5643 RNAs (Supplementary Table S5). These sites showed the characteristic stop codon-proximal distribution previously reported for m^6^A and were found in many RNAs that encode proteins with known synaptic function and neurite localization (Figure 5B; Supplementary Figure S5B). We also observed a high degree of overlap between methylated RNAs identified by DART-seq and those identified by MeRIP-seq (6), indicating the accuracy of the DART-seq approach (Supplementary Figure S5C).

We next used Bullseye to identify differentially methylated sites and RNAs in the soma and neurite fractions. To do this, we examined C2U editing rates (%C2U), which correlate with m^6^A abundance (35,44). We found that average %C2U values were generally higher for transcripts isolated from neurites compared to those from the soma (Figure 5C), and a large proportion of RNAs had elevated levels of methylation in neurites compared to the soma (Figure 5D). In fact, we identified a total of 1366 RNAs with increased m^6^A (higher %C2U) in neurites and only 136 RNAs with increased m^6^A in the soma (Figure 5D and E; Supplementary Figure S5D; Supplementary Table S5). All of these transcripts were detected in both the soma and neurites, suggesting that the methylation differences were not due to RNA enrichment in one fraction over the other, but instead reflect compartment-specific m^6^A signatures. Additionally, consistent with what we observed when analyzing MeRIP-seq data (Figure 3C), we found that RNAs with a greater number of m^6^A sites were more likely to have reduced neurite enrichment in *Mettl3* KO cells (Supplementary Figure S5E). We also found that RNAs exhibiting increased editing in neurites were enriched for genes with known synaptic localization and function (Supplementary Figure S5E). Interestingly, the *Camk2a* and *Map2* mRNAs were edited at significantly higher levels in neurites compared to the soma (Figure 5D and E), which is consistent with a role of m^6^A in promoting the localization of these transcript to neurites.

To determine whether m^6^A sites in distinct regions of an mRNA are associated with subcellular mRNA localization, we examined the distribution of m^6^A within mRNAs from the soma and neurites. We found that m^6^A sites in neurite-enriched mRNAs were more biased toward the 3′UTR compared to non-enriched or soma-enriched mRNAs (Figure 5F). The 3′UTR bias of m^6^A in neurite-enriched mRNAs was also observed when examining MeRIP-seq (6,22) and m^6^A-CLIP (24) datasets (Figure 5F). Interestingly, *cis*-acting elements contributing to mRNA localization are most often found in 3′UTRs (3,80,81). Thus, the observed enrichment of m^6^A within the 3′UTRs of neurite-localized transcripts may reflect m^6^A acting as an additional *cis*-acting element capable of promoting the localization of some mRNAs to neurites. Altogether, our data suggest that a subset of methylated mRNAs contains elevated levels of m^6^A in neurites and that m^6^A sites are abundant within 3′UTRs of neurite-enriched transcripts.

### YTHDF proteins regulate the neurite localization of *Camk2a* and *Map2* mRNAs

We next sought to understand the mechanism through which m^6^A controls mRNA localization in neurons. The functional roles of m^6^A in mRNA are largely mediated by m^6^A reader proteins (12). The most well-studied readers are the YTHDF proteins, which bind directly to m^6^A via their conserved YTH domain. There are three YTHDF proteins (YTHDF1,2, and 3), all of which are localized primarily to the cytoplasm (14,87–89). In non-neuronal cells, YTHDF proteins have been shown to influence the subcellular localization of mRNAs, including their recruitment to P-bodies and stress granules (14,17,21). Within neurons, these proteins are abundant within dendrites and synapses (22). Therefore, the YTHDF proteins are well-positioned to mediate m^6^A-dependent mRNA localization in neurons.

**Figure 6.**
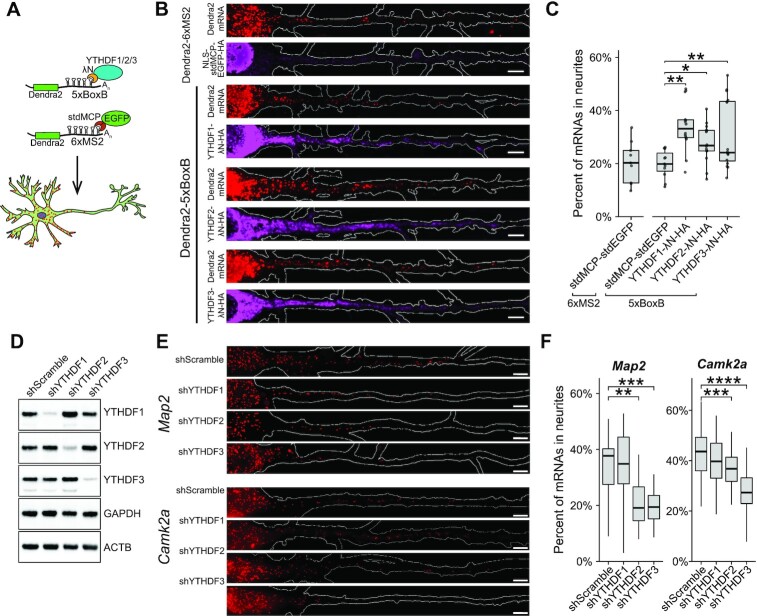
m^6^A reader proteins YTHDF2 and YTHDF3 regulate mRNA localization to neurites. (**A**) Schematic representation of mRNA tethering experiments. Hippocampal neurons were transduced with a lentivirus expressing either Dendra2-5xBoxB or Dendra2-6xMS2 and with a lentivirus expressing NLS-HA-stdMCP-stdEGFP or YTHDF1/2/3-λN-HA. Sequential immunostaining for the HA tag and smFISH for Dendra2 was done at DIV7. (**B**) Tethering of YTHDF proteins to a reporter mRNA increases mRNA localization to neurites. Representative images for cells expressing Dendra2-6xMS2 or Dendra2-5xBoxB reporter mRNA together with YTHDF1/2/3-λN-HA proteins or stdMCP-EGFP-HA (control). Dendra2 smFISH signal is shown in red; anti-HA immunofluorescence is shown in purple. Neuron outline is shown in white. Scale bars, 5 μm. (**C**) Boxplots of the quantification of reporter mRNA localization as measured by smFISH. The identity of the reporter (6xMS2 = Dendra2-6xMS2; 5xBoxB = Dendra2-5xBoxB) and the protein co-expressed with the reporter are indicated. Individual values are plotted as gray dots. (**D**) Western blot shows knockdown of YTHDF proteins in cultured hippocampal neurons after 7 days of expression of the indicated shRNA. (**E**) Representative images of endogenous *Map2* (upper panels) and *Camk2a* (lower panels) smFISH in hippocampal neurons expressing the indicated shRNAs. Neuron outline is shown in white. Scale bars, 5 μm. (**F**) Boxplots of the quantification of the percentage of *Map2* and *Camk2a* mRNAs in the neurites of hippocampal neurons after expression of the indicated shRNAs. A two-way Wilcoxon-test corrected for multiple comparison was used to test significance: *P < 0.05, **P < 0.01, ***P < 0.001, ****P < 0.0001. See also Supplementary Figure S6.

To determine whether YTHDF proteins are involved in m^6^A-dependent mRNA localization, we used the BoxB/λN-based system (90) to individually tether each of the YTHDF proteins to the 3′UTR of a Dendra2-encoding reporter mRNA in cultured hippocampal neurons (Figure 6A). As a negative control, we also generated a similar reporter using the MS2/MCP system (91,92) to tether stdMCP-stdEGFP to the Dendra2 3′UTR (Figure 6A). Overexpression of YTHDF1-,2-, or 3-λN fusion proteins together with Dendra2-5xBoxB mRNA enabled robust recruitment of the YTHDF proteins to the Dendra2 mRNA as measured by RNA immunoprecipitation (RIP)-RT-qPCR (Supplementary Figure S6A and B). This interaction was not observed when YTHDF1-, 2- or 3-λN was co-expressed with Dendra2-6xMS2 mRNA, indicating the specificity of the tethering system (Supplementary Figure S6B). We then expressed YTHDF1-, 2- or 3-λN together with Dendra2-5xBoxB mRNA in hippocampal neurons and detected Dendra2 mRNA molecules by smFISH (Figure 6B, Supplementary Figure S6C). Tethering of YTHDF1 or YTHDF3, but not YTHDF2, led to an increase in reporter mRNA expression when compared to tethering of stdMCP-stdEGFP or to the untethered reporter mRNA alone (Supplementary Figure S6D). Additionally, tethering of any of the three YTHDF proteins led to a small but significant increase in the percentage of mRNAs localization to neurites (median ± SD, stdMCP: 19.9 ± 5.2%, YTHDF1: 33.2 ± 13.7%, YTHDF2: 27.1 ± 11.2%, YTHDF3: 24.2 ± 13.0%) (Figure 6C; Supplementary Figure S6E). These results suggest that YTHDF1, 2 and 3 are individually capable of promoting neuritic mRNA localization.

We next asked whether endogenous YTHDF proteins in neurons promote the localization of mRNAs to neurites. To this end, we knocked down each *Ythdf* mRNA in cultured hippocampal neurons using lentiviral-mediated delivery of shRNAs together with CFP to identify transduced neurons. Expression of YTHDF shRNAs led to robust and specific loss of each individual YTHDF protein (Figure 6D and Supplementary Figure S6F). We then used smFISH to assess the subcellular localization of the *Camk2a* and *Map2* transcripts, both of which we had found to exhibit m^6^A-dependent neurite enrichment (Figure 3E and F; Figure 5C). Interestingly, we found that depletion of YTHDF2 and YTHDF3, but not YTHDF1, caused a reduction in *Camk2a* and *Map2* localization to neurites, despite the fact that all three proteins bound to the *Camk2a* and *Map2* transcripts in hippocampal neurons (Figure 6E and F; Supplementary Figure S6G and H).

YTHDF1 had the most robust impact on reporter mRNA localization in tethering experiments (Figure 6C). We therefore wondered whether the lack of effect of YTHDF1 on *Camk2a* and *Map2* localization was due to functional compensation by YTHDF2/3, as has been demonstrated for all three YTHDF proteins in other contexts (15,16,93). However, we did not observe an increase in YTHDF2 or YTHDF3 levels following YTHDF1 depletion (Figure 6D). Moreover, double knockdown of YTHDF1/2 or YTHDF1/3 did not exacerbate the effects of YTHDF2 or YTHDF3 depletion on mRNA localization (Supplementary Figure S6I). However, double knockdown of YTHDF2/3 caused a more robust decrease in mRNA localization to neurites than knockdown of either YTHDF2 or YTHDF3 alone, an effect which was not further enhanced by triple knockdown of YTHDF1/2/3 (Supplementary Figure S6I). Together, these data suggest a unique role for YTHF2 and YTHDF3, but not YTHF1, in mediating the neurite localization of *Camk2a* and *Map2* mRNAs.

### YTHDF proteins bind to overlapping and distinct transcripts in hippocampal neurons

**Figure 7.**
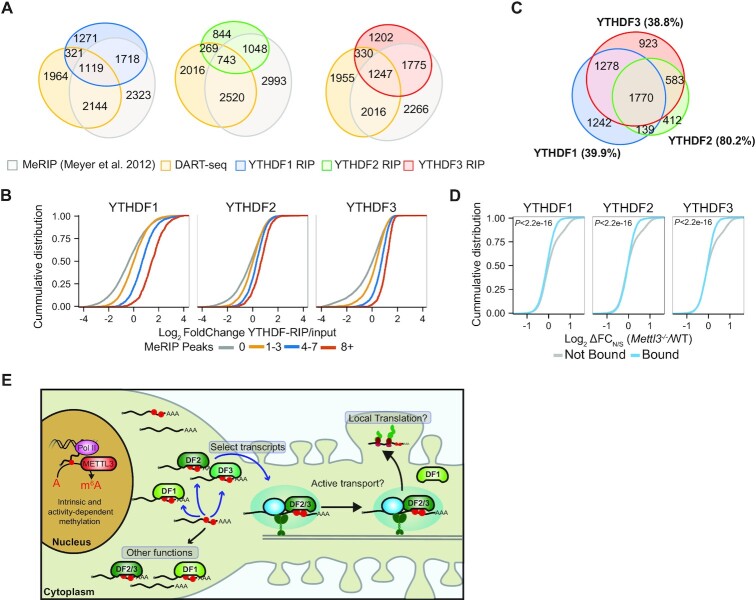
YTHDF proteins bind overlapping and distinct RNAs in neurons. (**A**) Euler diagrams showing overlap of enriched RNAs in YTHDF1, YTHDF2 or YTHDF3 RIP-seq libraries with DART-seq and MeRIP-seq (6) datasets. (**B**) Cumulative distribution plots of the enrichment in YTHDF RIP over input for transcripts with 0, 1–3, 4–7 or >8 MeRIP-seq peaks. (**C**) Euler diagram showing overlap of bound RNAs in YTHDF1, YTHDF2, and YTHDF3 RIP-seq libraries**. (D)** Cumulative distribution plots of the changes in neurite enrichment (ΔFC_N/S_) in *Mettl3* KO neurons for transcripts bound or not by YTHDF1/2/3. Included are RNAs with baseMean ≥250. *P* value is the result of a Kolmogorov–Smirnov test. (**E**) Model for the role of m^6^A in promoting mRNA localization to neurites. Select mRNAs are methylated in the nucleus and are recognized by YTHDF proteins. Binding of YTHDF2 or YTHDF3 to a subset of synaptic transcripts promotes their localization to neurites. This process may involve YTHDF2/3-mediated recruitment of mRNAs to RNA transport granules. See also Supplementary Figure S7.

To better understand how YTHDF proteins interact with RNAs in hippocampal neurons, we performed RIP-seq to uncover the target transcripts of endogenous YTHDF1, 2 and 3 transcriptome-wide (Supplementary Figure S7A). Consistent with the known role of these proteins as m^6^A readers, we found a high degree of overlap between YTHDF target RNAs and methylated transcripts identified by MeRIP-seq (6) and DART-seq (Figure 7A), and we observed a positive correlation between the number of MeRIP-seq peaks (6) and the enrichment of RNAs in each RIP sample relative to input (Figure 7B). Additionally, RNA targets of all three YTHDF proteins shared similar GO category associations, which included those related to synapse function and localization and chromatin remodeling (Supplementary Figure S7B).

We found that most of the RNAs bound by any individual YTHDF protein were also bound by at least one other YTHDF protein, and that nearly 40% of all bound transcripts were common targets of all three YTHDF proteins (Figure 7C). The finding that all three YTHDF proteins share many common target RNAs in neurons is similar to what has been reported in other cell types (15). However, we also identified hundreds of RNAs that were enriched exclusively in YTHDF1, 2 or 3 RIP datasets, suggesting that a subset of RNAs may be bound uniquely by specific YTHDF proteins (Figure 7C; Supplementary Figure S7C–F; Supplementary Table S6).

We next examined whether the target RNAs of YTHDF proteins exhibit m^6^A-dependent neurite enrichment. Nearly 20% of neurite-enriched RNAs were bound by YTHDF proteins (Supplementary Figure S7G), and the target RNAs of all three YTHDF proteins were more likely to exhibit reduced neurite enrichment in *Mettl3* KO neurons compared to non-target RNAs (Figure 7D). Interestingly, the transcripts that were uniquely bound by each individual YTHDF protein showed similar levels of overlap with neurite-enriched RNAs (Supplementary Figure S7G), as well as with RNAs exhibiting reduced neurite enrichment in *Mettl3* KO neurons (Supplementary Figure S7H), suggesting that no single YTHDF protein binds preferentially to transcripts destined for neurites.

Altogether, these data demonstrate that YTHDF proteins share many common RNA targets in hippocampal neurons, but that individual YTHDF proteins also bind to some transcripts uniquely. Moreover, YTHDF proteins bind to a subset of neurite-enriched RNAs, many of which encode proteins important for synaptic function. Collectively, our data support a model in which m^6^A influences the localization of a subset of neuronal mRNAs to neurites (Figure 7E). We find that for the *Camk2a* and *Map2* mRNAs, this effect is mediated by the YTHDF2 and YTHDF3 reader proteins. However, it is also likely that YTHDF1 facilitates the localization of other RNAs to neurites based on its ability to direct neurite localization in tethering experiments. Given the emerging roles of YTHDF proteins and m^6^A in neurodevelopment and learning and memory, our findings suggest that m^6^A-mediated subcellular mRNA localization may be an important mechanism through which the epitranscriptome impacts brain function.

## DISCUSSION

Neurons possess a complex architecture which often includes an elaborate dendritic tree and a long axon that can extend hundreds of microns from the cell body. The sensing and processing of signaling events at these distal locations requires rapid responses to ensure the maintenance and strengthening of synaptic connections. Such responses are mediated by a host of intricate regulatory mechanisms which collectively enable the local translation of mRNAs residing in remote locations such as synapses and the growth cone. A critical component of this complex regulatory process is the transport of select mRNAs to distal neuronal compartments. Several sequence motifs and structural elements within mRNAs have been implicated in mRNA localization, but the full complement of *cis*-acting elements that control this process is far from being elucidated. Here, we identify adenosine methylation as an additional layer of regulation which influences the subcellular localization of neuronal mRNAs. Our studies uncover hundreds of neuronal transcripts whose targeting to neurites is altered following knockout of the m^6^A methyltransferase METTL3, and we provide the first direct demonstration that m^6^A residues within the 3′UTR promote the neuritic localization of a subset of neuronal transcripts.

m^6^A provides a versatile mechanism for regulating the subcellular localization of a transcript of interest within the cell. Since most, if not all, mRNAs contain the core m^6^A consensus (RAC) within their sequence, virtually any mRNA is susceptible to m^6^A methylation. Furthermore, subsets of m^6^A sites appear to be targeted by m^6^A demethylating enzymes, and this mechanism has been proposed to mediate dynamic regulation of m^6^A within the brain (7,94–98). However, we did not observe widespread KCl-dependent changes in mRNA localization, suggesting that activity-dependent m^6^A changes may not lead to a substantial increase in mRNA localization to neurites. Rather, we find that the effects of m^6^A on mRNA localization are more likely to impact mRNA localization at steady-state.

Our analysis of m^6^A from soma and neurite compartments of hippocampal neurons revealed 1,144 RNAs containing elevated m^6^A levels in the neurite-enriched fraction. However, our data from *Mettl3* KO neurons indicate that not all methylated mRNAs exhibit m^6^A-dependent neurite localization, and loss of m^6^A does not result in the complete removal of any transcript from neurites. This is consistent with previous studies indicating that nearly all m^6^A sites are sub-stoichiometric, with many present at relatively low levels (44,99,100). Thus, it is likely that m^6^A is just one of many factors that controls the subcellular localization of neuronal transcripts.

Two mRNAs that we focused on specifically in this study were *Map2* and *Camk2a*, both of which encode proteins that play important roles in synaptic plasticity and maintenance (82–85). We find that these transcripts localize to neurites in a *Mettl3*-dependent manner, and we identify individual m^6^A sites within the 3′UTRs of these transcripts that are required for localization. Previous studies have identified 3′UTR elements in both these mRNAs that promote their dendritic localization (85,101–105). Although it is possible that m^6^A residues within these elements may contribute to localization, none of the m^6^A sites that we mutated in our studies were within these regions. Therefore, our results suggest that m^6^A contributes to localization independently of these other dendritic targeting elements. Future studies dissecting the unique contributions of 3′UTR sequence elements, structural features, and m^6^A sites to mRNA localization will help illuminate the degree to which these *cis-*acting factors enhance localization and whether they work in a coordinated fashion in the recruitment of *trans*-acting factors. Additionally, localization elements have been identified in 5′UTRs and CDSs (62,106–109), which are also susceptible to methylation, so it will be interesting to explore whether m^6^A outside of the 3′UTR contributes to transcript localization. Indeed, the *Shank1* 3′UTR did not respond in our m^6^A mutant reporter assay, so it is possible that m^6^A residues within the 5′UTR or CDS of this transcript may contribute to the effects on *Shank1* localization that we observed following *Mettl3* depletion.

Our experiments using both tethering assays and knockdown of endogenous proteins have provided mechanistic insights into how m^6^A controls mRNA localization in neurons. We find that the m^6^A reader proteins YTHDF2 and YTHDF3 contribute to endogenous *Map2 and Camk2a* neurite localization in cultured neurons and that their binding to a reporter mRNA 3′UTR can induce its localization to neurites. In contrast, depletion of YTHDF1 does not impact the neurite localization of *Map2* and *Camk2a*, despite the fact that tethering of YTHDF1 to a reporter mRNA enables its neurite localization. Our data show that YTHDF1 binds to *Map2* and *Camk2a* in neurons, so the lack of effect of YTHDF1 on the localization of these transcripts is unlikely to be due to reduced recognition of these mRNAs. Indeed, transcriptome-wide, we observe that all three YTHDF proteins share many common target RNAs in neurons. One possibility is that YTHDF1 acts downstream of YTHDF2 and YTHDF3 to control the local translation of methylated mRNAs in neurons. Consistent with this idea, YTHDF1 contributes to activity-dependent translation in hippocampal neurons (25) and is required for the translation, but not the localization, of the *Apc* mRNA in dendrites (22). Of course, it remains possible that YTHDF1 does contribute to mRNA localization in neurons but that it only carries out this function for a subset of its target mRNAs.

Surprisingly, we did not observe an effect of loss of *Mettl3* on target mRNA stability (Supplementary Figure S3E), in contrast to the known role of m^6^A and YTHDF proteins on mRNA destabilization in other cell types (14–16,93). In fact, we observed an increase in mRNA abundance when tethering YTHDF1 or YTHDF3 to a reporter mRNA. It is notable that previous studies investigating m^6^A in the brain have failed to find a positive correlation between the loss of *Mettl3* (110), *Mettl14* (111), or YTHDF1 (25) and changes in the abundance of methylated mRNAs. It is possible that YTHDF proteins play an additional role in neurons to promote the long-distance transport of target mRNAs, instead of inducing degradation. This is an attractive model, given that distally-localized mRNAs within the brain have longer half-lives than non-localized transcripts (57), presumably because they must be stabilized in order to travel long distances. Consistent with this idea, YTHDF2 has been identified as one of 65 proteins enriched in Stau2-containing RNA transport granules (112). Therefore, it is possible that YTHDF2 recruits m^6^A-containing mRNAs to RNA transport granules, enabling these transcripts to be selectively trafficked to distal compartments (Figure 7E). It will be interesting to explore this possibility in future studies and to determine whether YTHDF3 operates through a similar mechanism. Indeed, all three YTHDF proteins contain low-complexity regions which facilitate their association with RNA granule structures, such as P-bodies and stress granules (14,17,21).

In addition to the YTHDF proteins, other RBPs may mediate m^6^A-dependent mRNA localization in neurons. For instance, FMRP and MARTA1 promote dendritic localization of the *Camk2a* and *Map2* mRNAs, respectively (70,113). It was recently proposed that FMRP and the MARTA1 homolog KHSRP preferentially bind m^6^A-containing RNA (19,114). However, these proteins bind to many sequences outside of the canonical m^6^A DRACH motif (63,115,116), and the increased affinity of FMRP for methylated RNA over unmethylated RNA is small (∼2.5-fold or even negligible for some sequences (19,117)). In contrast, the YTHDF m^6^A reader proteins show a much greater preferential binding to m^6^A-containing RNA over unmodified RNA (10- to 100-fold) (14,118,119). However, it is certainly possible that other m^6^A readers may be mediating m^6^A-dependent RNA localization, and ultimately the roles of distinct readers are likely to be transcript-specific.

The delivery of select transcripts to distal neuronal compartments enables the local protein synthesis events which contribute to synaptic strengthening. Our data indicate a transcript-specific role for m^6^A in controlling mRNA localization, but the impact of m^6^A on local translation is currently unknown. Previous studies have linked m^6^A to translation control through multiple mechanisms (25,87,120,121), and studies in dorsal root ganglion neurons have shown a specific role for m^6^A residues within the *Gap43* mRNA in regulating its local translation in response to growth factors (97). Future studies using global m^6^A depletion will likely shed more light on the scope of this effect and whether m^6^A is a widely used mechanism for the regulation of local protein synthesis in neurons. Additionally, both *Map2* and *Camk2a* undergo local translation in dendrites to promote long-term potentiation and synaptic plasticity (82–85). It will therefore be interesting to explore whether reduced dendritic localization of their mRNAs contributes to the defects in synapse morphology and function observed when m^6^A readers, writers, or erasers are depleted from hippocampal neurons (7,22,25,110).

## DATA AVAILABILITY

Bullseye is an open-source pipeline for analysis of DART-seq data and is available in the GitHub repository: https://github.com/mflamand/Bullseye.

The data that support the findings of this study have been deposited in NCBI’s Gene Expression Omnibus under accession code **GSE194208**.

## Supplementary Material

gkac251_Supplemental_Files
