# Supplementary material for: m6A and YTHDF proteins contribute to the localization of select neuronal mRNAs
Source: Nucleic Acids Res. 2022 Apr 19;50(8):4464–83. doi: 10.1093/nar/gkac251 (PMC9071445; doi:10.1093/nar/gkac251)
Supplement: gkac251_Supplemental_Files [file gkac251_supplemental_files.zip › Supplementary information_revisions.pdf]

Supplementary tables description

Supplementary Table 1. Relative RNA enrichment in neurite of hippocampal neurons

1. Results of DESeq2 analysis measuring neurite enrichment in hippocampal neurons (baseMean ≥ 10).
2. List of RNAs enriched in neurites ($FC_{N/S}$ ≥ 1.5, FDR ≤ 0.05)
3. List of RNAs enriched in soma ($FC_{N/S}$ ≥ 1.5, FDR ≤ 0.05)

Supplementary Table 2. Relative changes in RNA abundance following 2h of KCl treatment in hippocampal neurons.

1. Results of DESeq2 analysis measuring differential expression in the soma of neurons following 2h KCl treatment (baseMean ≥ 10).
2. List of RNAs with increased abundance following 2h KCl treatment ($FC_{N/S}$ ≥ 2, FDR ≤ 0.05)
3. List of RNAs with decreased abundance following 2h KCl treatment ($FC_{N/S}$ ≥ 2, FDR ≤ 0.05)

Supplementary Table 3. Relative changes in neurite enrichment following KCl treatment.

1. Results of DESeq2 analysis for neurite enrichment under KCl treatment. List contains all genes.
2. List of RNAs enriched in neurites under KCl treatment ($FC_{N/S}$ ≥ 1.5, FDR ≤ 0.05)
3. List of RNAs enriched in soma under KCL treatment ($FC_{N/S}$ ≥ 1.5, FDR ≤ 0.05)
4. Results of Xtail analysis measuring differential enrichment in neurites and soma following membrane depolarization with KCl for 2 h, list contains all genes.:
   - log2FC_NS_v1 and pvalue_v1 are calculated by the analysis of the difference in fold change in RNA abundance in each compartment across conditions.
   - log2FC_NS_v2 and pvalue_v2 by the analysis of the difference in the ratio of RNA abundance in neurites and soma ($log2Ratios_{N/S}$) in sample pairs across conditions.
   - The final pvalue_final and log2FC_NS_final is selected as the least significant (highest p-value) of v1 and v2 results.
5. List of RNAs with increased neurite enrichment following 2 h of KCl treatment (ΔFC_{NS} final ≥ 1.5, pvalue_final ≤ 0.05)
6. List of RNAs with decreased neurite enrichment following 2 h of KCl treatment (ΔFC_{NS} final ≤ -1.5, pvalue_final ≤ 0.05)

Supplementary Table 4. Relative changes in RNA abundance and neurite enrichment in *Mettl3* KO neurons.

1. Results of DESeq2 analysis measuring differential expression in the soma of *Mettl3* KO neurons (baseMean ≥ 10). List contains all genes.
2. List of RNAs with increased abundance in *Mettl3* KO neurons ($FC_{N/S}$ ≥ 1.5, FDR ≤ 0.05).
3. List of RNAs with decreased abundance in *Mettl3* KO neurons ($FC_{N/S}$ ≤ -1.5, FDR ≤ 0.05).
4. Results of Xtail analysis measuring differential enrichment in neurites and soma of Mettl3 KO neurons. This list contains all genes, including genes with decreased enrichment in neurites which were not statistically significant. For example, see *Camk2a*, *Shank1*, *Ddn.*
   - log2FC_NS_v1 and pvalue_v1 are calculated by the analysis of the difference in fold change in RNA abundance in each compartment.

- log2FC_NS_v2 and pvalue_v2 by the analysis of the difference in the ratio of RNAs in neurites and soma ($log2Ratios_{N/S}$) in sample pairs across genotype.
- The final pvalue_final and log2FC_NS_final is selected as the least significant (highest p-value) of v1 and v2 results.

5. List of RNAs with increased enrichment in neurites in *Mettl3* KO neurons (ΔFC_{NS} final >= 1.5, pvalue_final ≤ 0.05).
6. List of RNAs with decreased enrichment in neurites in *Mettl3* KO neurons (ΔFC_{NS} final >= -1.5, pvalue_final ≤ 0.05).
7. List of abundant RNAs (baseMean ≥ 250) with at least 20% decrease in neurite enrichment in *Mettl3* KO neurons (no *p*-value filtering). The number of m^6A sites identified by DART-seq, MeRIP (1,2), m6A-CLIP/IP(3) and YTHDF1 iCLIP peaks(4) is indicated for each gene.

Supplementary Table 5. Lists of m^6A sites identified by DART-seq in hippocampal neurons.

1. List of all called m^6A sites.
    - Listed are the genomic positions, name of the gene and strand.
    - WT_avg and KO_avg are the average %C2U editing in all WT or *Mettl3* KO samples respectively.
    - Soma_avg and Neurite_avg are the average %C2U editing in all soma or neurite samples respectively. Each contains samples from both Ctrl and KCl-treated samples in the respective compartment.
2. List of methylated genes and editing score across compartments and conditions
    - The score is the sum of the average editing at all called sites in each gene.
    - Log2FoldChange for each condition are obtained from the glm fitted data.

Supplementary Table 6. List of RNAs bound by in YTHDF1, YTHDF2 and YTHDF3 in hippocampal neurons.

1. Result of DESeq2 analysis for RNA enrichment in YTHDF RIP over input.
    - All transcripts with baseMean >= 10 are shown.
    - The log2FoldChange.YTHDF# and p.adj.YTHDF# are the columns representing the enrichment and P-values obtained by DESeq2 for comparing each YTHDF RIP to input samples.
2. List of RNAs bound by YTHDF1 ($FC_{N/S}$ ≥ 1.25, FDR ≤ 0.05)
3. List of RNAs uniquely bound by YTHDF1
4. List of RNAs bound by YTHDF2 ($FC_{N/S}$ ≥ 1.25, FDR ≤ 0.05)
5. List of RNAs uniquely bound by YTHDF2
6. List of RNAs bound by YTHDF3 ($FC_{N/S}$ ≥ 1.25, FDR ≤ 0.05)
7. List of RNAs uniquely bound by YTHDF3

Supplementary Table 7. List of primers, data for FISH quantification, and FISH probe sets used in this study.

1. Data and statistics for FISH imaging quantification in Figures 3F, 4B, 6C, 6F and Supplementary Figures S6E,S6I.
2. Genomic coordinates of m^6A sites mutated in the reporter assay

3. Primers used for cloning, mutagenesis, and qPCR.
4. List of plasmids used in this study.
5. Sequences of FISH probes used in this study.

Supplementary figures

Supplementary Figure S1

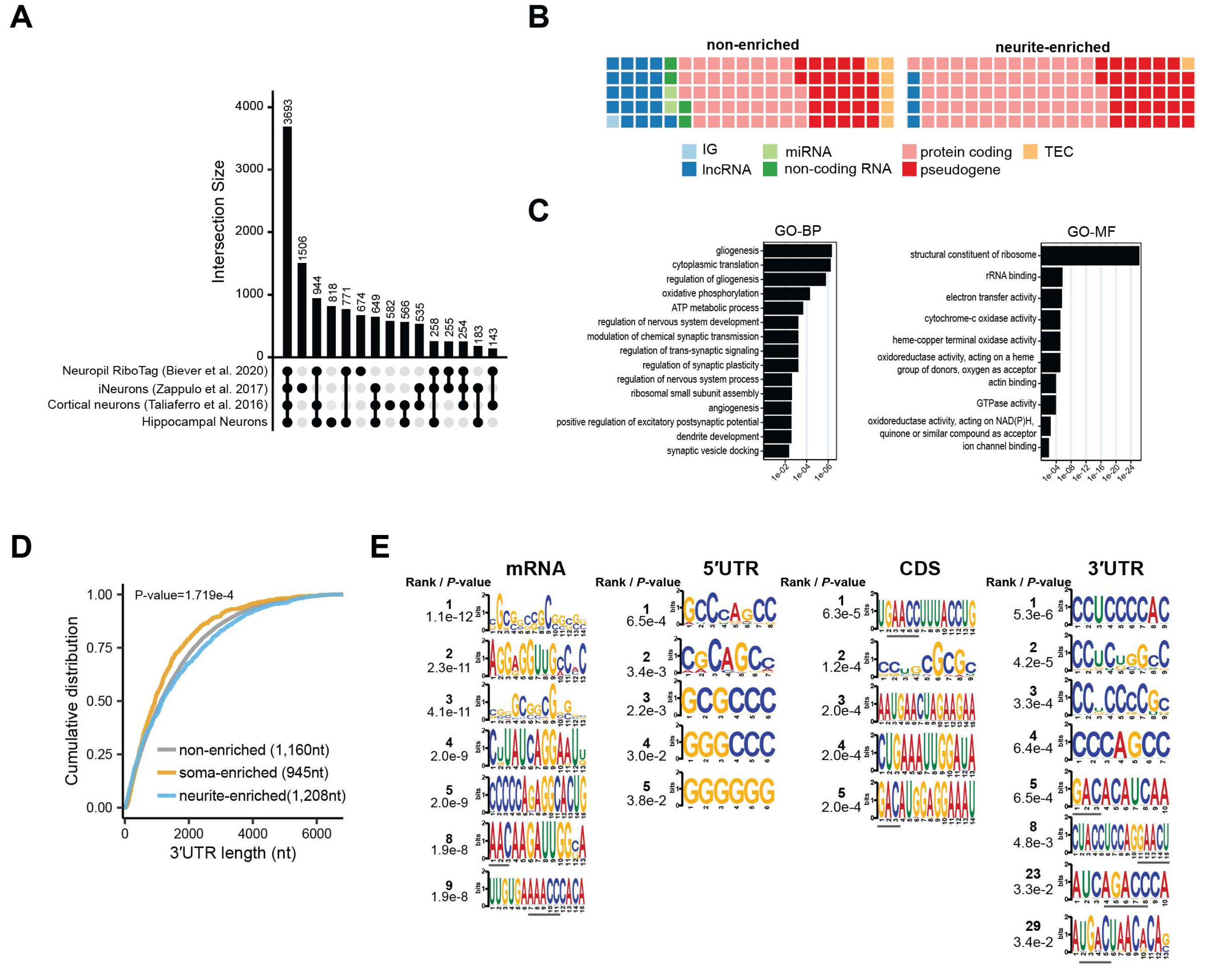

Supplementary Figure 1: Characterization of the local hippocampal neuron transcriptome. Related to Figure 1. **A)** Upset plots overlap of genes expressed (>= 5 FPKM or >= 5 RPKM) in the neurite of hippocampal neurons (this study), neurites of iNeurons (5), hippocampal neuropil (6) or cortical neurons (7). **B)** Waffle chart charts showing the distribution of GENCODE biotypes of non-enriched transcripts (left) and neurite-enriched transcripts (right). Each square represents 1 %. IG: Immunoglobulin, lncRNA: long non-coding RNA, TEC: To be Experimentally Confirmed. **C)** False discovery rate for the top 15 overrepresented biological processes (BP) and molecular function (MF) gene ontology terms for neurite enriched RNAs. **D)** Cumulative distribution of 3′UTR length of neurite-enriched and soma-enriched mRNAs compared to non-enriched mRNAs. The 3′UTR median length of mRNAs in each category is indicated in parenthesis. *P*-value was calculated as the result of a two-sided Kolmogorov-Smirnov test between neurite-enriched and non-enriched mRNAs. **E)** The top 5 most enriched motifs found in sequences from mRNA, 5′UTR, CDS, or 3′UTR regions of neurite-enriched mRNAs. Occurrence of RAC and DRACH-containing motifs (m^6A consensus sequence) are underlined in gray. Additional motifs identified in mRNAs (ranked 8th and 9th) and in the 3′UTRs (ranked 8th, 23rd and 28th) containing DRACH motifs are also shown. *P* values for individual motif enrichment were calculated using Fisher's exact test.

Supplementary Figure S2

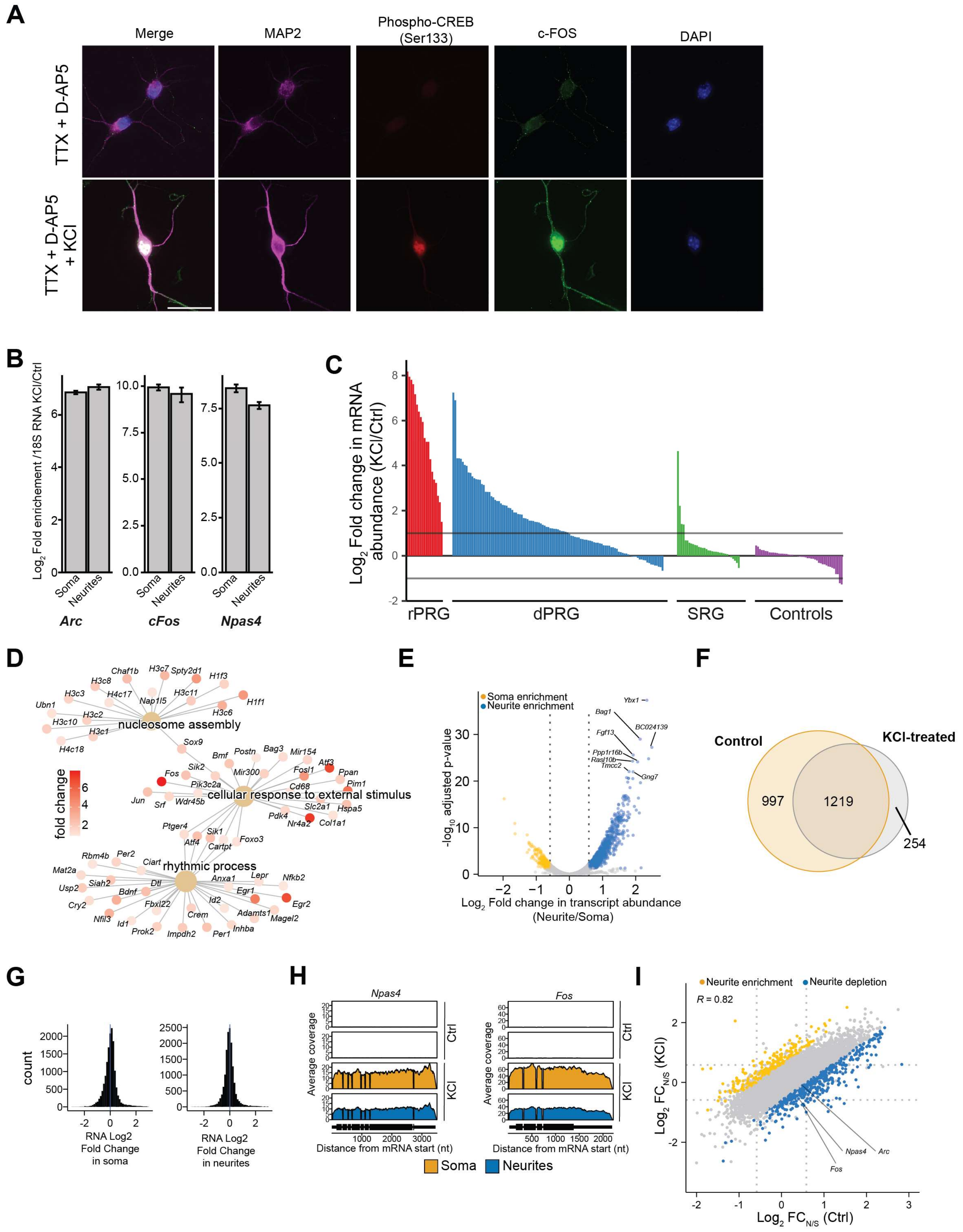

Supplementary Figure S2: Characterizing the local transcriptome of KCl-treated neurons. Related to Figure 2. **A)** Immunofluorescence shows induction of c-FOS and CREB Ser133 phosphorylation in hippocampal neurons treated with KCl. Uninduced cells (top) and induced cells (bottom) are shown. Scale bars, 25 µm. **B)** Quantitative RT-PCR (qRT-PCR) shows increased expression of *Arc*, *Fos*, and *Npas4* in the soma and neurites following KCl treatment for 2 hours. Expression of each transcript is normalized to 18S rRNA. N=3, error bars; standard deviation. **C)** Membrane depolarization induces expression of IEGs. Change in mRNA abundance of rapid and delayed primary response genes (rPRG, dPRG), secondary response genes (SRG), and control genes(8) in neurons treated with KCl for 2 hours. **D)** Gene network of the top enriched biological process gene ontology terms following KCl-treatment. **E)** Volcano plot shows transcripts enriched and depleted in hippocampal neurites following KCl treatment. *Blue,* neurite-enriched RNAs (FC ≥ 1.5, FDR ≤ 0.05). *Yellow*, Neurite-depleted RNAs (FC ≤ 1.5, FDR <0.05). The top five most enriched RNAs with the lowest p-values are labeled. **F)** Euler diagram showing RNAs that are enriched in neurites of hippocampal neurons in Control (D-AP5 and TTX treated) and KCl-treated neurons. **G)** Histogram showing the distribution of Log2 Fold change in RNA abundance in soma and neurites following KCl-treatment. **H)** Gene coverage tracks for the highly induced genes *Npas4* and *Fos* in soma and neurites fractions following KCl-treatment. Both genes show lower coverage in neurite than in the soma. **I)** Scatterplot of the changes in neurite enrichment ($FC_{N/S}$) in Ctrl and KCl-treated neurons. *Yellow*: RNAs enriched in neurites ($FC_{N/S}$ ≥ 1.5, p ≤ 0.05), *Blue*: Genes depleted from neurites ($FC_{N/S}$ ≤ -1.5, p ≤ 0.05). The highly induced IEGs *Fos*, *Npas4* and *Arc* are labeled. Pearson correlation for $FC_{N/S}$ in Ctrl and KCl-treated neurons is indicated. Dotted lines: ±Log2(1.5).

Supplementary Figure S3

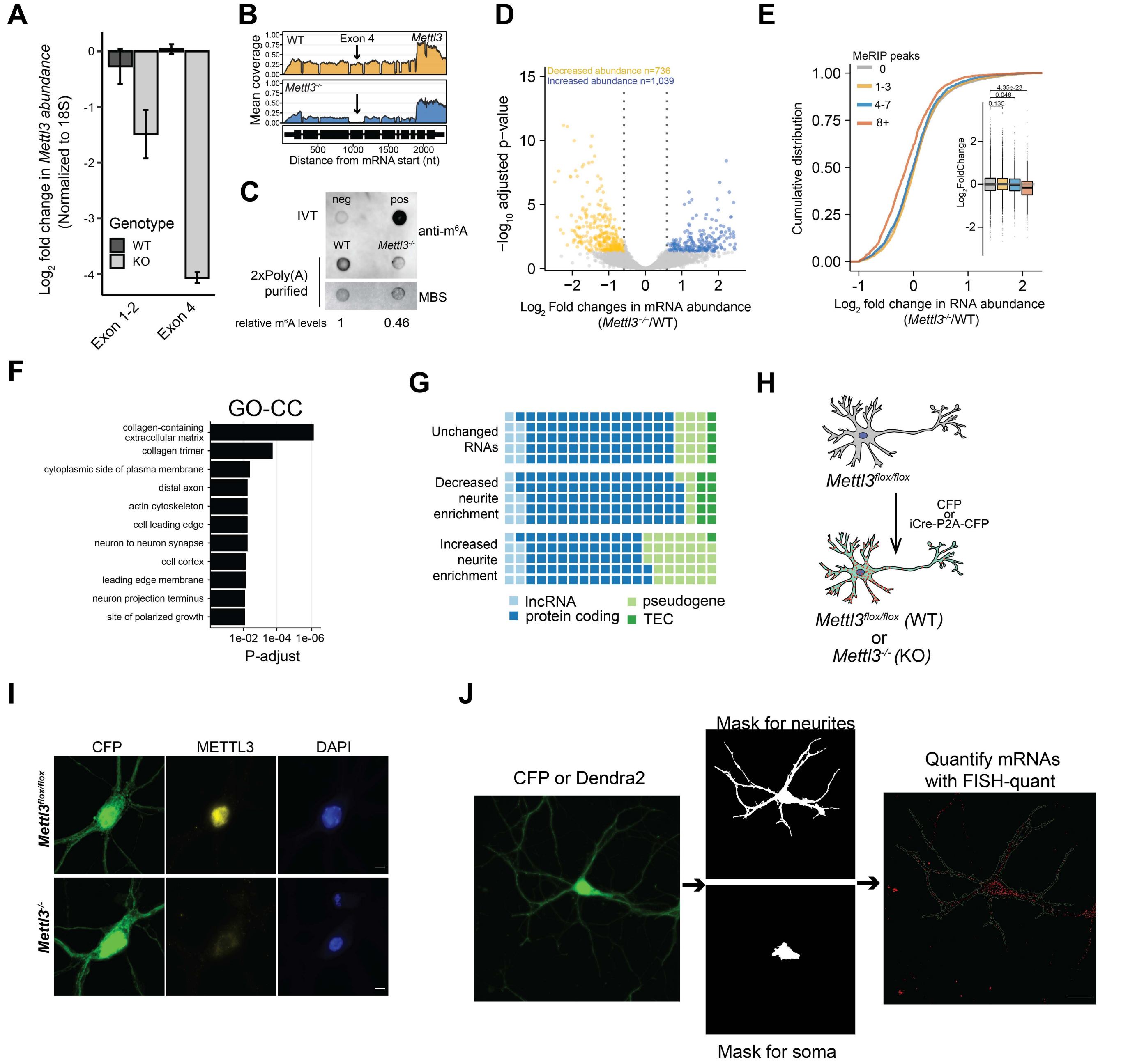

Supplementary Figure S3: Gene expression and local transcriptome changes in *Mettl3* knockout neurons. Related to Figure 3.

A) qRT-PCR shows relative expression of *Mettl3* exon1-2 or exon4 at DIV15 in WT or *Mettl3*$^{flox/flox}$ hippocampal neurons transduced with AAV expressing Cre recombinase. N=3; Data is represented as the mean ± S.D. **B),** Coverage tracks of *Mettl3* in the soma of WT and *Mettl3*$^{-/-}$ neurons show the loss of exon 4 flanked by loxP sites. **C)** m^{6}A dot blot of 100 ng of poly(A)-purified RNA from WT or Mettl3$^{-/-}$ neurons. m^{6}A levels relative to RNA amount are indicated. IVT: In vitro transcribed RNA with 0 or 100% m^{6}A. MBS: Methylene blue staining of RNA on membrane. **D)** Volcano plot showing changes in mRNA abundance in *Mettl3*$^{-/-}$ neurons. *Yellow*: RNAs with decreased expression (FC ≤ -1.5, p ≤ 0.05), *Blue*: RNAs with increased expression ($FC_{N/S}$ ≥ 1.5, p ≤ 0.05). **E)** Cumulative distribution plots and boxplots (insets) showing

the fold change in mRNA abundance relative to the number of MeRIP-seq peaks (1). **F)** False discovery rate for the top overrepresented cellular compartment gene ontology terms for RNAs with decreased neurite enrichment in *Mettl3*$^{-/-}$ cells. **G)** Waffle chart charts showing the distribution of GENCODE biotypes of unchanged, decreased, or increased neurite decreased neurite enrichment in *Mettl3*$^{-/-}$ neurons. Each square represents 1 %. lncRNA: long non-coding RNA, TEC: To be Experimentally Confirmed. **H)** Strategy for detecting endogenous mRNA localization by smFISH. Hippocampal neurons isolated from *Mettl3*$^{flox/flox}$ mice expressing CFP or iCre-P2A-CFP were used in smFISH experiments at DIV14. **I)** Immunofluorescence staining shows loss of METTL3 protein in *Mettl3*$^{-/-}$ neurons. Top row: *Mettl3*$^{flox/flox}$ neurons transduced with AAV-hSyn1-CFP. Bottom row: *Mettl3*$^{flox/flox}$ neurons transduced with AAV-hSyn1-Cre-P2A-CFP (*Mettl3*$^{-/-}$). **J)** Pipeline for detection and quantification of mRNA by smFISH. For each image, a mask of individual cells is created from the CFP or Dendra2 signal. A mask for the cell body is then manually defined. The percentage of RNAs localizing to neurites is then quantified as the total number of RNAs identified outside the soma divided by the total number of RNAs in each cell.

Supplementary Figure S4

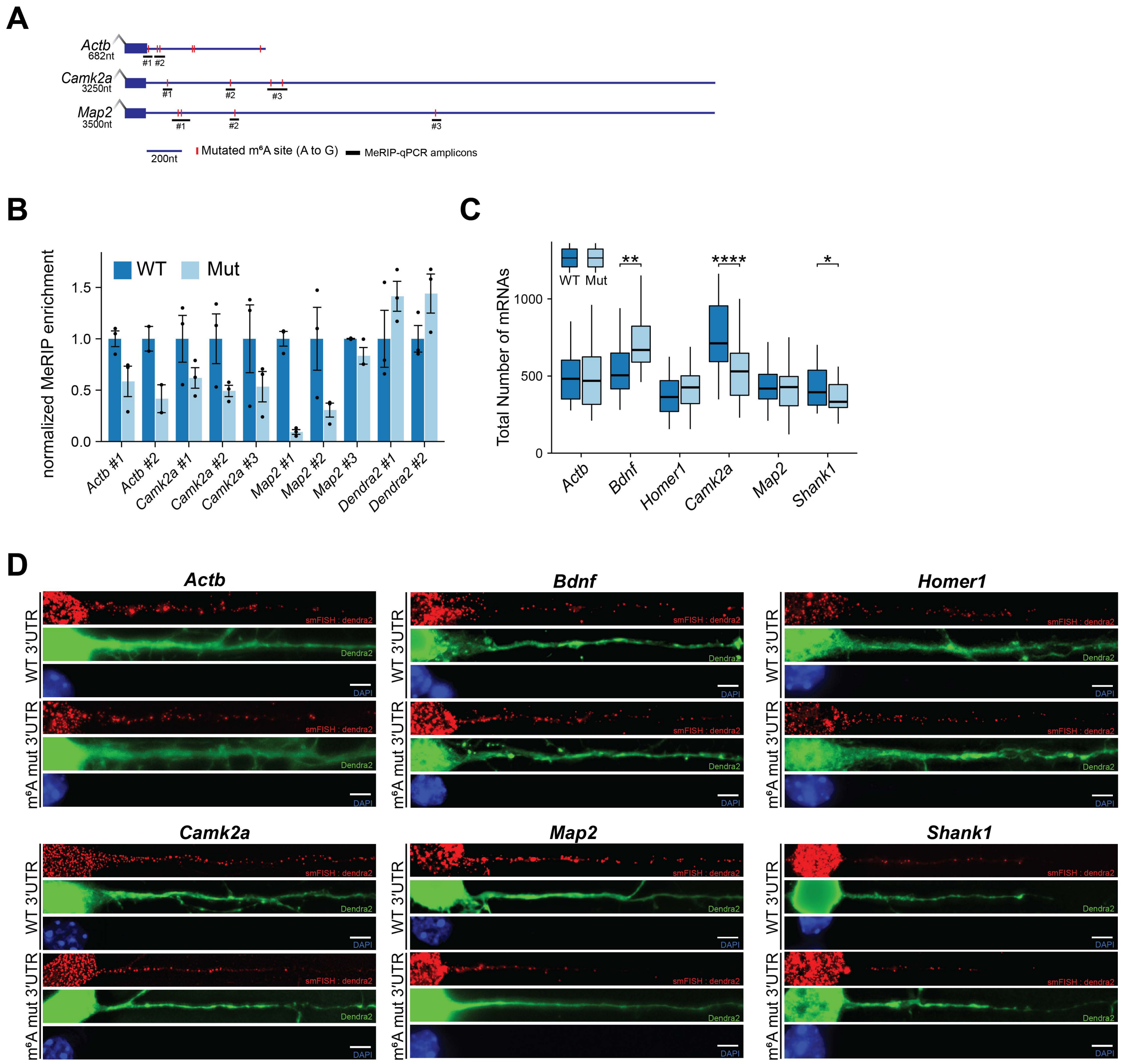

Supplementary Figure S4: smFISH detecting reporter mRNAs containing candidate 3'UTRs. Related to Figure 3. **A)** Shown are the *Actb*, *Camk2a* and *Map2* 3′UTRs cloned downstream of Dendra2 in reporter mRNAs and positions of m^{6}A sites mutated in each reporter (red). The length of each 3'UTR and the number of mutations is indicated for each reporter. The complete list of mutated positions can be found in Supplementary Table 7. The positions of amplified fragments for MeRIP-qPCR in (B) are shown for each reporter. **B)** Mutation of m^{6}A sites in the 3′UTR of *Actb*, *Camk2a* and *Map2* reporter mRNAs reduces m^{6}A levels. MeRIP-qRT-PCR in HEK293T cells expressing the indicated reporter mRNA shows relative enrichment of WT or m^{6}A mutant reporters normalized to immunoprecipitation efficiency**.** Reporters contain all m^{6}A mutations. Dendra2 #1 and Dendra2 #2 probes correspond to invariable regions in the Dendra2 CDS in both WT and m^{6}A mutant reporters. Each datapoint is normalized to the mean enrichment of the WT reporter. Data are displayed as the mean +/- SE. n= 3 biological replicates. Individual replicates are indicated as black dots. **C)** Boxplots of the quantification of smFISH data showing the total number of total Dendra2 mRNAs found in each

cell for each reporter. A two-sided Wilcoxon-test corrected for multiple comparison was used to test for significance: $^{*}P<0.05$, $^{**}P<0.01$, $^{***}P<0.001$. **D)** Representative images showing smFISH staining for each reporter mRNA. Images show Dendra2 smFISH (top), DENDRA2 fluorescent protein (middle) and DAPI (bottom). Dendra2 smFISH images for *Actb*, *Camk2a,* and *Map2* 3′UTR reporters are the same as those in Figure 4. Scale bars, 5 µm.

Supplementary Figure S5

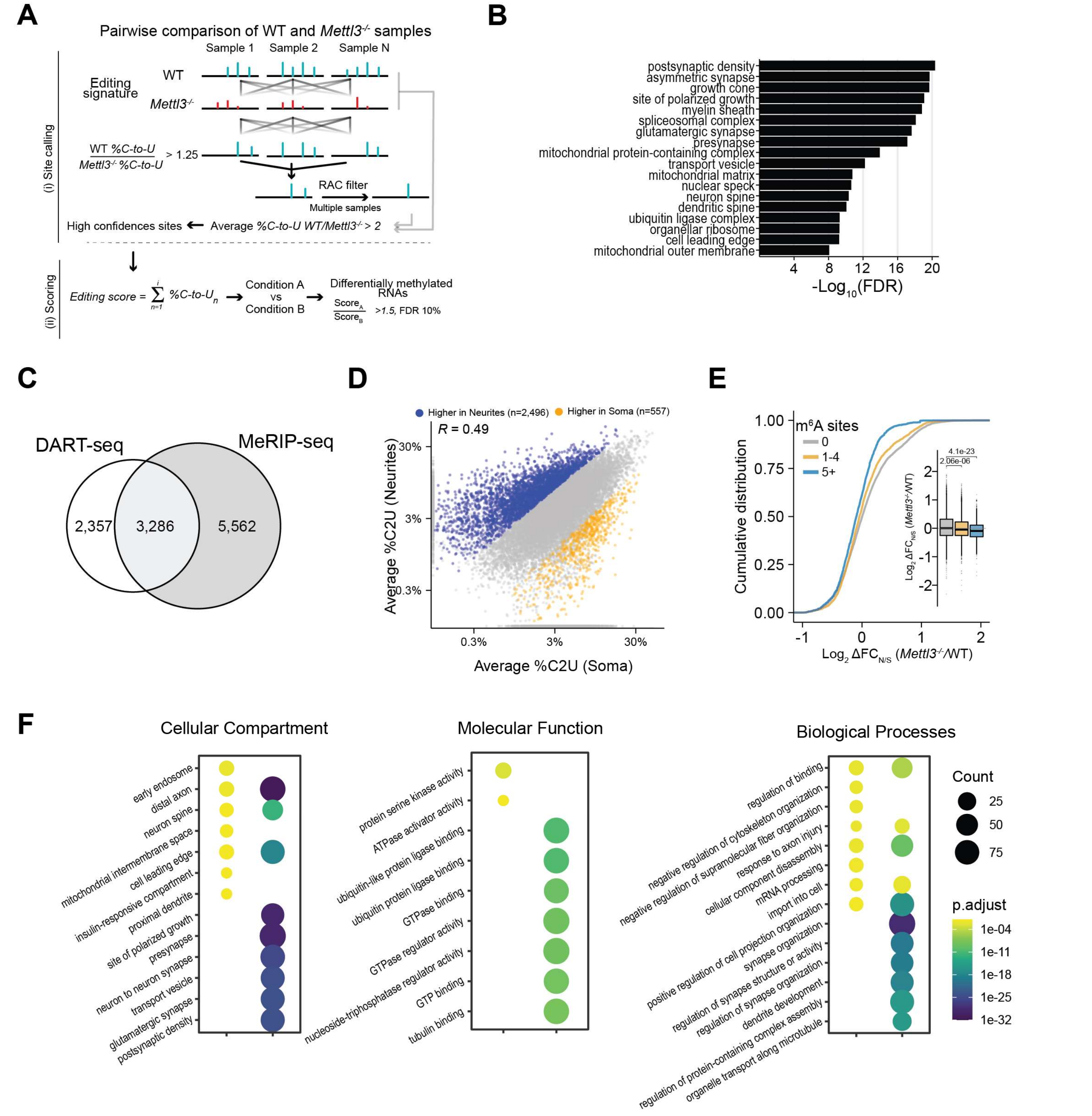

Supplementary Figure S5: Gene expression and local transcriptome changes in *Mettl3* knockout neurons. Related to Figure 5.

A) Overview of the Bullseye analysis pipeline for detection of m^{6}A sites. (i) In the first step, sites are identified in each biological replicate which contain ≥3 C2U mutations, %C2U editing of 2-95%, and at least 1.25-fold more editing (%C2U) compared to each individual *Mettl3* KO sample. Sites found in at least 2 replicates and in the RAC motif are kept for analysis. The final high confidence list of sites is generated by keeping sites for which %C2U values are on average at least 1.5-fold greater in all WT samples compared to all *Mettl3* KO samples. (ii) In the second step, differentially methylated RNAs can be identified across conditions. For each condition, RNAs are assigned an "editing

score" based on the cumulative average %C2U value in all high confidence sites. Differentially methylated RNAs are then identified as those with a >1.5-fold increased editing score between conditions. **B)** m^6A sites are enriched in genes with synaptic localization. Shown are the top enriched cellular compartment gene ontology terms for RNAs containing m^6A sites compared to all expressed genes. **C)** Euler plot showing the overlap of m^6A-containing mRNAs detected by DART-seq and MeRIP-seq in whole brain (1). **D)** Scatterplot of the average %C2U editing at single sites in soma or neurite samples. Sites that are edited to a higher degree in neurites or soma (fold change %C2U ≥ 2, FDR ≤ 0.05) are colored in blue and yellow, respectively. Pearson correlation of %C2U is indicated. **E)** Cumulative distribution plots and boxplots (insets) of the changes in neurite enrichment ($\Delta FC_{N/S}$) in *Mettl3*$^{-/-}$ neurons for transcripts with 0, 1-4, or more than 5 m^6A sites identified by DART-seq. Included are RNAs with baseMean≥250; n=7,070 (0 sites); n=4,010 (1-4 sites); n=1258 (5+ sites). *P*-value is the result of a two-sided Mann-Whitney U-test. F) Gene ontology enrichment analysis of mRNAs with increased or decreased %C2U at the RNA level in soma and neurite fractions. Shown are the top enriched terms for cellular compartment, biological process, and molecular function gene ontology categories for each group. The set of all genes containing DART-seq sites was used as the background.

Supplementary Figure S6

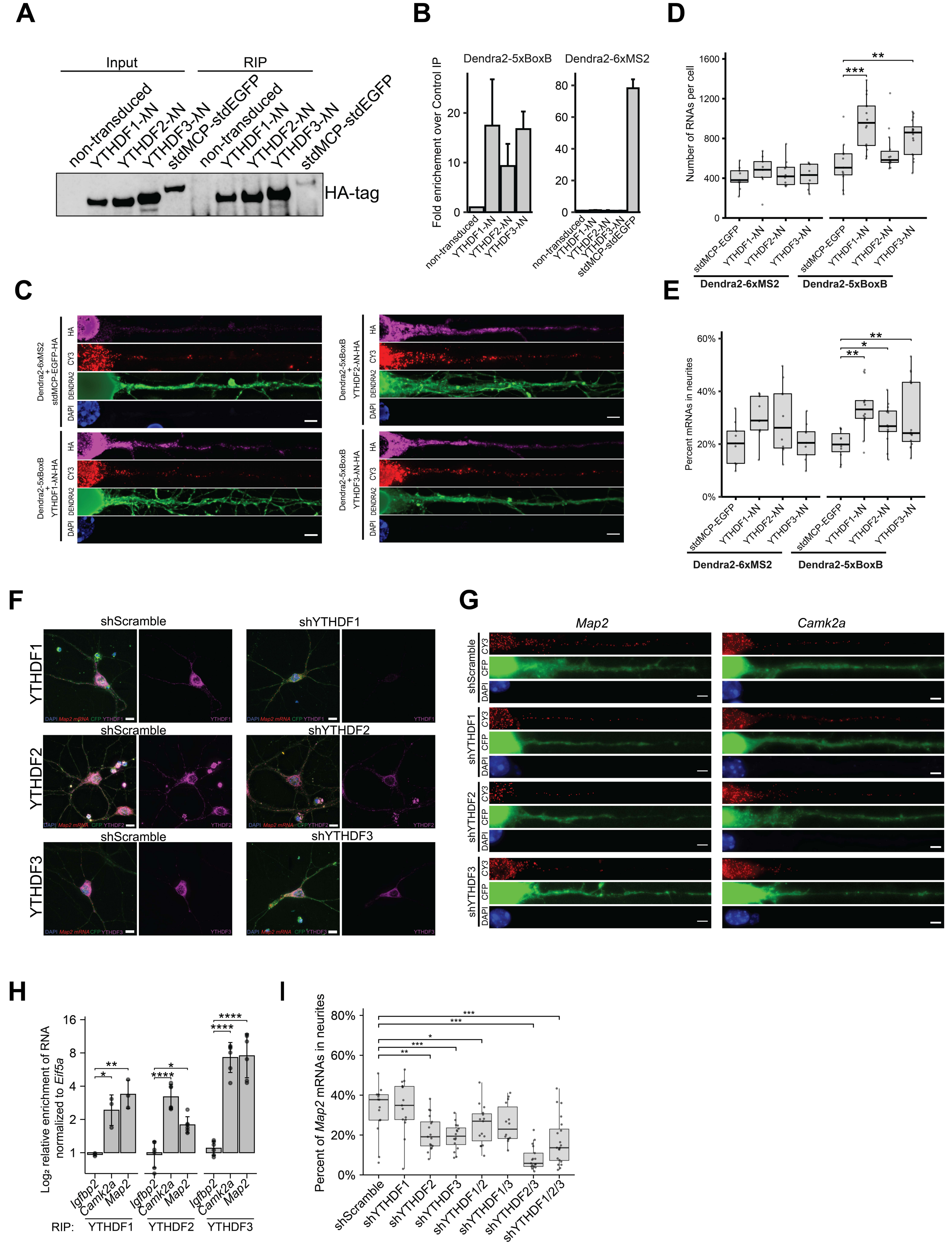

Supplementary Figure 6: m^{6}A reader proteins control mRNA *Map2* and *Camk2a* localization. Related to Figure 6. **A)** HA-tagged proteins are efficiently immunoprecipitated in anti-HA RNA immunoprecipitation (RIP) experiments. Western blot analysis of anti-HA RIP performed in HEK293T cells. Images are representative of 3 biological replicates. **B)** RT-qPCR analysis shows that the Dendra2-5xBoxB mRNA is co-immunoprecipitated with each YTHDF-λN protein and that Dendra2-6xMS2 is co-immunoprecipitated with NLS-HA-stdMCP-stdEGFP, N=3. Error bars: standard deviation. Enrichment is normalized to the amount of HA tagged protein immunoprecipitated as measured by western blot in **(A)**. **C)** Representative images of neurons expressing Dendra2-6xMS2 mRNA and NLS-HA-stdMCP-stdEGFP (top 2 images), or Dendra2-5xBoxB mRNA and each YTHDF-λN protein (bottom 6 images). Images for Dendra2 smFISH and HA IF are the same as those shown in Figure 6B. Scale bars, 5 μm. **D)** Tethering of YHTDF1 or YTHDF3 results in an increased number of mRNAs in primary neurons. Shown is the quantification of mRNA abundance of each reporter and protein pair by smFISH. **E)** Quantification of neurite localization by smFISH in RNA tethering experiments is shown. Tethering of YHTDF proteins to the Dendra2 mRNA increases its localization to neurites (Dendra2-5xBoxB mRNA, right), whereas the localization of a non-tethered mRNA is not affected by expression of YTHDF proteins (Dendra2-6xMS2, left). Individual values are plotted as gray dots. A two-way Wilcoxon-test was used to test significance: **P*<0.05, ***P*<0.01, ****P*<0.001. **F)** Validation of YTHDF protein knockdown**.** Representative images of immunofluorescence (IF) staining of YTHDF1, YTHDF2 or YTHDF3 in hippocampal neurons transduced with virus expressing a scramble shRNA (left 2 columns) or shRNA targeting each YTHDF protein (right 2 columns). Images are composites of DAPI (blue), Map2 smFISH (red), CFP (green) and YTHDF IF (purple). The immunofluorescence signal alone is also shown. Scale bars, 10 μm. **G)** Representative images of endogenous *Map2* and *Camk2a* smFISH in primary neurons expressing the indicated shRNAs. Images show *Map2* smFISH (top), CFP (middle) and DAPI (bottom). Images of the *Map2* smFISH channel are the same one shown in Fig. 6. Scale bars, 5 μm. **H)** RIP-RT-qPCR analysis shows that YTHDF proteins bind to *Camk2a* and *Map2*. *Igfbp2* is shown as a control mRNA. n=5. Relative enrichment over non-methylated mRNA *Eif5a* is displayed as the mean +/- S.D. Individual values for each biological replicate are indicated as grey dots. A two-way Wilcoxon-test: **P*<0.05, ***P*<0.01, *****P*<0.00001. **I)** Boxplots of the quantification of the percentage of *Map2* mRNAs in the neurites of hippocampal neurons after expression of the indicated shRNAs. A two-way Wilcoxon-test was used to test significance: **P*<0.05, ***P*<0.01, ****P*<0.001.

Supplementary Figure S7

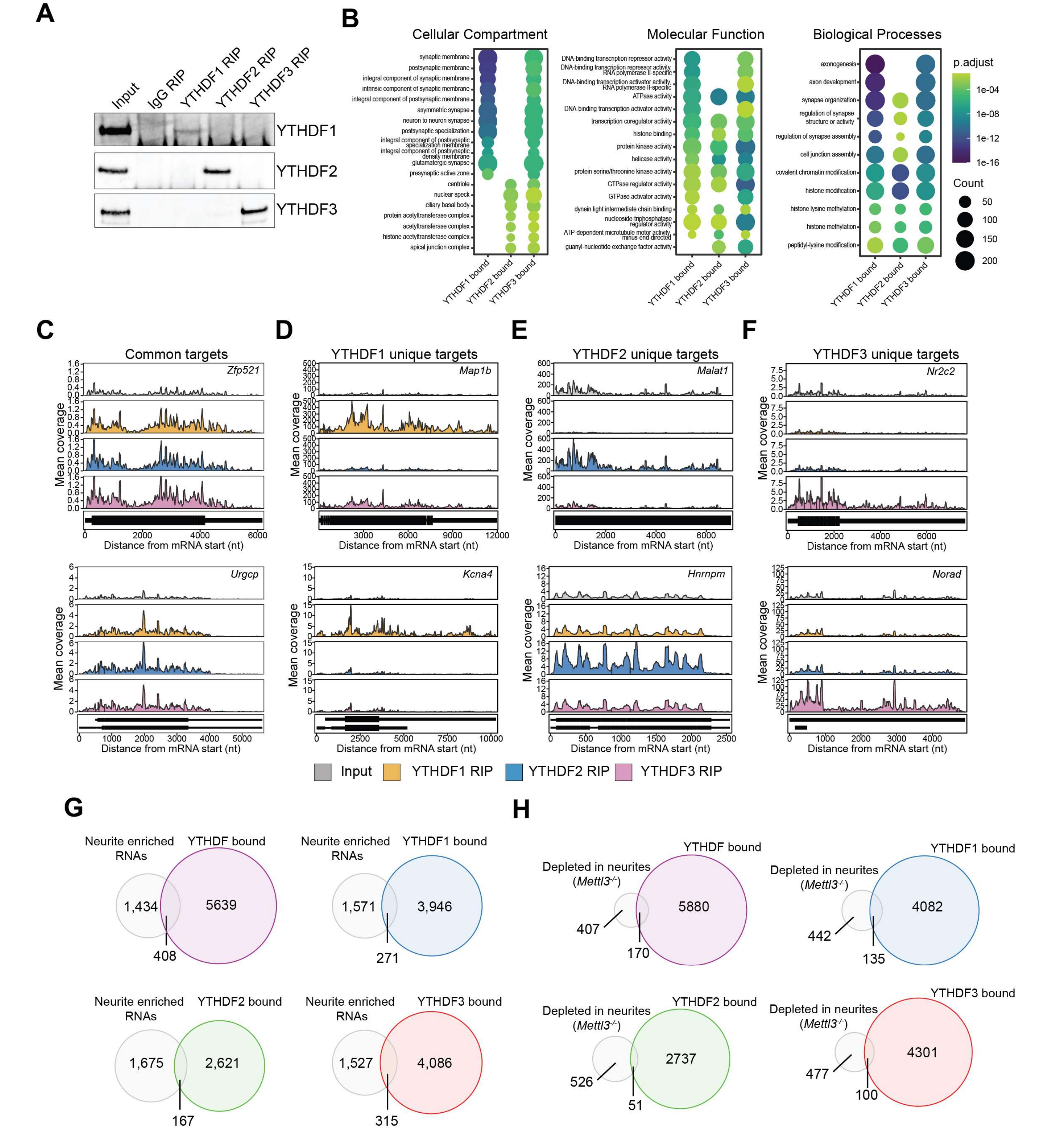

Supplementary Fig. 7: YTHDF proteins bind to overlapping and distinct target RNAs in hippocampal neurons. Related to Fig. 7. a, Endogenous YTHDF proteins are specifically immunoprecipitated in cultured neurons. Western blot analysis of each YTHDF protein following immunoprecipitation. Images are representative of 3 biological replicates. **b,** Top enriched cellular compartment, biological process, and molecular function gene ontology terms for RNAs bound by YTHDF1, YTHDF2, or YTHDF3. YTHDF1 bound, n=3,921; YTHDF2 bound, n=2,630; YTHDF3 bound, n=4,214. **c-f,** Example coverage tracks for RNAs bound by all YTHDF proteins (**c**), or uniquely by YTHDF1 (**d**), YTHDF2 (**e**), or YTHDF3 (**f**). **g,** Euler diagrams showing overlap of RNAs enriched in the neurites of hippocampal neurons and RNAs

bound by any YTHDF protein (top left), or YTHDF1, YTHDF2, or YTHDF3. **h,** Euler diagrams showing overlap of RNAs depleted from the neurites of *Mettl3* KO neurons and transcripts bound by any YTHDF protein (top left), or YTHDF1, YTHDF2, or YTHDF3.

Supplementary references

1. Meyer, K.D., Saletore, Y., Zumbo, P., Elemento, O., Mason, C.E. and Jaffrey, S.R. (2012) Comprehensive analysis of mRNA methylation reveals enrichment in 3' UTRs and near stop codons. *Cell*, **149**, 1635-1646.
2. Merkurjev, D., Hong, W.T., Iida, K., Oomoto, I., Goldie, B.J., Yamaguti, H., Ohara, T., Kawaguchi, S.Y., Hirano, T., Martin, K.C. *et al.* (2018) Synaptic N(6)-methyladenosine (m(6)A) epitranscriptome reveals functional partitioning of localized transcripts. *Nat Neurosci*, **21**, 1004-1014.
3. Ke, S., Alemu, E.A., Mertens, C., Gantman, E.C., Fak, J.J., Mele, A., Haripal, B., Zucker-Scharff, I., Moore, M.J., Park, C.Y. *et al.* (2015) A majority of m6A residues are in the last exons, allowing the potential for 3' UTR regulation. *Genes Dev*, **29**, 2037-2053.
4. Shi, H., Zhang, X., Weng, Y.L., Lu, Z., Liu, Y., Lu, Z., Li, J., Hao, P., Zhang, Y., Zhang, F. *et al.* (2018) m(6)A facilitates hippocampus-dependent learning and memory through YTHDF1. *Nature*, **563**, 249-253.
5. Zappulo, A., van den Bruck, D., Ciolli Mattioli, C., Franke, V., Imami, K., McShane, E., Moreno-Estelles, M., Calviello, L., Filipchyk, A., Peguero-Sanchez, E. *et al.* (2017) RNA localization is a key determinant of neurite-enriched proteome. *Nat Commun*, **8**, 583.
6. Biever, A., Glock, C., Tushev, G., Ciirdaeva, E., Dalmay, T., Langer, J.D. and Schuman, E.M. (2020) Monosomes actively translate synaptic mRNAs in neuronal processes. *Science*, **367**.
7. Taliaferro, J.M., Vidaki, M., Oliveira, R., Olson, S., Zhan, L., Saxena, T., Wang, E.T., Graveley, B.R., Gertler, F.B., Swanson, M.S. *et al.* (2016) Distal Alternative Last Exons Localize mRNAs to Neural Projections. *Mol Cell*, **61**, 821-833.
8. Tyssowski, K.M., DeStefino, N.R., Cho, J.H., Dunn, C.J., Poston, R.G., Carty, C.E., Jones, R.D., Chang, S.M., Romeo, P., Wurzelmann, M.K. *et al.* (2018) Different Neuronal Activity Patterns Induce Different Gene Expression Programs. *Neuron*, **98**, 530-546 e511.
